# Lunar Regolith Simulant–Filled High-Performance Thermoplastic Composites for Lunar Construction and Their Potential for Additive Manufacturing

**DOI:** 10.3390/polym18192371

**Published:** 2026-09-29

**Authors:** Ivan P. Storozhuk, Alexander G. Khina, Denis P. Bulkatov, Thant K. Phone, Sergey V. Reznik, Elizaveta N. Abramova, Ilya V. Tretyakov, Nikolay S. Malyshev, Semen Yu. Fedorov

**Affiliations:** 1 NTI Center “Digital Materials Science: New Materials and Substances”, Bauman Moscow State Technical University, Moscow 105005, Russia; storozhukip@bmstu.ru (I.P.S.); bulkatov@bmstu.ru (D.P.B.); tretyakov.i@bmstu.ru (I.V.T.); malyshev.ns@bmstu.ru (N.S.M.); fedorovs@bmstu.ru (S.Y.F.); 2 Special Mechanical Engineering Faculty, Bauman Moscow State Technical University, Moscow 105005, Russia; xp22ma034@student.bmstu.ru (T.K.P.); sreznik@bmstu.ru (S.V.R.); abramova_en@bmstu.ru (E.N.A.)

**Keywords:** lunar construction, lunar regolith simulant, high-performance thermoplastics, polymer composites, polyetherimide, polyphenylsulfone, hot pressing, melt processing, additive manufacturing, 3D-printing

## Abstract

The use of lunar regolith (LR) for lunar construction is a key route to reducing the mass of materials transported from Earth. In this work, highly filled composites based on polyphenylsulfone (PPSU) with T_g_ = 219.1 °C and modified polyetherimide (PEI) with T_g_ = 247.1 °C containing up to 92.5 wt.% of LR simulant were prepared by dry blending the LR simulant and the polymer powders, followed by hot pressing. Their internal structure, mechanical, thermal, thermomechanical, and melt/flow properties were evaluated. The properties of the composites strongly depended on the LR simulant content and were governed by two competing mechanisms: restriction of polymer-chain mobility and of matrix deformation by the rigid mineral filler at moderate LR simulant contents, and increased porosity and loss of matrix continuity caused by binder deficiency at excessive filler loadings. At LR simulant contents of up to 60 wt.% for PPSU and 85 wt.% for PEI, dense, low-porosity (<10 vol.%) monolithic composites were formed, whereas higher filler loadings led to binder deficiency and increased porosity. LR simulant incorporation increased Shore D hardness from 75 to 88 for PPSU- and from 86 to 96 for PEI-based composites. Maximum compressive strength/modulus reached 122 MPa/6.3 GPa for PPSU- and 193 MPa/7.4 GPa for PEI-based composites, compared with 93 MPa/2.3 GPa and 150 MPa/3.3 GPa for neat polymers, respectively. Thermal conductivity increased to 0.64 and 0.72 W/(m·K), while CLTE decreased to 14.2 × 10^−6^ K^−1^ and 10.1 × 10^−6^ K^−1^ for PPSU- and PEI-based composites, respectively. Composites containing 50–60 wt.% of LR simulant retained melt/flow capability, indicating their potential suitability for processing by melt extrusion and additive manufacturing. Based on the obtained data, recommendations were formulated for application of the developed composites in melt-based 3D printing of complex-geometry parts and hot-pressed simple-shape construction elements.

## 1. Introduction

In recent years, the interest in lunar programs, driven by both national space agencies and a growing number of commercial companies, has markedly increased [1,2,3,4]. Unlike the Apollo missions, which were limited to short-duration expeditions to relatively accessible near-equatorial regions of the Moon, NASA’s Artemis program, along with other contemporary lunar initiatives, is aimed at multiple landings and the development of long-term lunar surface infrastructure for sustained human presence and subsequent utilization of local resources [5,6]. However, the construction of such infrastructure is constrained by the high cost of transporting materials and equipment from Earth, which, according to published techno-economic estimates, is currently on the order of USD 1 million per kg and, even under optimistic forecasts, may decrease only to USD 100–300 thousand per kg within the next 5–10 years [7]. This limitation is especially critical for construction materials intended for use on the Moon, as they are required in large quantities. Therefore, in situ resource utilization (ISRU), and in particular the use of lunar regolith (LR) as a construction raw material, has become one of the most actively developed approaches for reducing the mass of materials that must be transported from Earth for lunar construction [8,9,10,11].

Physically, LR is a loose, unconsolidated material produced by prolonged meteoroid bombardment and space-weathering processes on the lunar surface; it consists of mineral and rock fragments, breccia fragments, glasses of impact and volcanic origin, and agglutinates—aggregates of regolith grains welded together by micrometeoroid-impact melt and containing nanophase metallic iron [12]. The chemical composition of LR is dominated by SiO_2_, Al_2_O_3_, FeO, MgO, CaO, and TiO_2_, with SiO_2_ typically representing the most abundant component and accounting for approximately 40–50 wt.%. From a mineralogical standpoint, the crystalline fraction of LR is dominated by silicate minerals, primarily plagioclase, pyroxene, and olivine, with smaller amounts of ilmenite and other mineral phases [13]. However, LR is not compositionally uniform across the lunar surface, and two principal types are generally distinguished in ISRU: mare and highland regolith. Mare regolith is derived predominantly from basaltic rocks and contains higher proportions of pyroxene, olivine, and ilmenite, resulting in generally higher FeO and TiO_2_ contents. In contrast, highland regolith is associated mainly with anorthositic crust and is dominated by plagioclase, resulting in higher Al_2_O_3_ and CaO and lower FeO and TiO_2_ contents. Considerable compositional variability also occurs within both mare and highland regolith due to variations in local lithology and mixing of material redistributed by impact processes. Accordingly, LR simulants used in terrestrial experiments commonly include compositionally representative mare- or highland-type materials, although specialized simulants targeting particular physical, mechanical, or dust-related characteristics are also used [14].

Several main approaches to the use of LR as a construction raw material are currently being considered for lunar construction [15,16,17,18]. The least technologically demanding concepts involve the use of unconsolidated or mechanically compacted regolith to create protective embankments, surface layers, landing pads, and radiation-shielding covers [19,20]. A more technologically advanced approach involves thermal consolidation of the mineral raw material, including sintering, partial melting, or vitrification of regolith using microwave radiation, concentrated solar energy, or laser heating [21,22,23,24]. These methods enable the production of monolithic mineral elements without transporting large amounts of binder components from Earth; however, they usually require temperatures of approximately 800–1200 °C for sintering and up to 1100–1400 °C for localized melting, which is associated with high energy consumption and sensitivity of the process to the chemical, mineralogical, and particle-size composition of the regolith. An alternative group of methods includes technologies based on the introduction of inorganic or organic binders, including geopolymer systems, sulfur- and cement-like materials, as well as polymer composites [25,26]. In such systems, regolith particles are bound through the formation of a mineral or polymer matrix, which substantially reduces the processing temperature and expands the possibilities for shaping construction elements, including by casting, hot pressing, extrusion, and additive manufacturing. However, the key limitation of binder-based technologies remains the need to transport the binder from Earth or to organize its on-site production; therefore, the main challenge is to maximize the LR fraction while preserving the strength, integrity, and processability of the resulting materials.

Among binder-based approaches, LR/polymer composites represent a particularly promising class of materials because they combine the possibility of incorporating high fractions of LR with the formability of the polymer matrix and comparatively low-temperature processing [27,28,29,30,31]. In contrast to direct sintering or melting of regolith, polymer-based systems can be consolidated at temperatures governed by the softening, melting, or curing behavior of the binder rather than by mineral-phase sintering; for high-temperature thermoplastics, this typically corresponds to processing temperatures in the range of several hundred degrees Celsius. They can also be processed by hot pressing, extrusion, casting, or additive manufacturing, depending on the nature of the binder, its content, and the target geometry of the final part or component [32,33]. At the same time, the polymer binder remains a non-local component of the material system that must be transported from Earth. Therefore, the key materials-design challenge is to maximize the LR content while maintaining sufficient mechanical integrity, dimensional stability, and processability of the composite.

Thermoplastic binders are especially promising for this purpose. Compared with conventional thermoset composites, thermoplastic composites can provide higher toughness and improved resistance to cracking, brittle failure, and fatigue damage [34,35]. Furthermore, high-performance thermoplastics can exhibit better chemical resistance and stability under ionizing radiation, while some of them also show good resistance to ultraviolet exposure and environmental weathering [36,37,38]. Their processing does not require a prolonged irreversible curing stage, as they can be softened or melted upon heating and subsequently solidified upon cooling [39,40]. This reversible behavior enables reprocessing, recycling, repeated forming, and thermally assisted repair, while also making LR/thermoplastic composites compatible with melt-based manufacturing routes, including extrusion, fused filament fabrication (FFF), fused granular fabrication (FGF), and other material-extrusion approaches, which may facilitate future closed-loop manufacturing and in situ maintenance on the lunar surface [41]. One of the processing challenges associated with thermoplastic matrices, however, is their high melt viscosity, which can substantially hinder the uniform dispersion of filler particles, especially at high filler contents. This difficulty can be mitigated, for example, by preliminary grinding and dry blending of polymer and filler powders to obtain a homogeneous powder mixture, followed by melt processing or consolidation. Such an approach promotes a more uniform initial distribution of the filler before the polymer enters the highly viscous molten state. As a result, considerable attention has recently been paid to LR simulant/thermoplastic composites based on different polymer matrices, including polylactide (PLA) [42,43], polyethylene (PE) [44], polypropylene [45], polyimide and polyamide systems [33], as well as high-regolith-loaded poly(ether ether ketone) (PEEK), poly(ether ketone ketone) (PEKK), and composites for melt-based additive manufacturing and space infrastructure applications [46,47,48]. These studies demonstrate the growing interest in thermoplastic binders for lunar construction, ranging from low-temperature recyclable polymers to high-performance aromatic thermoplastics. Nevertheless, despite this increasing interest, most reported LR simulant/thermoplastic systems are still focused either on relatively low-temperature polymers or on a limited number of high-performance matrices, mainly the polyaryletherketone (PAEK) family, such as PEEK- and PEKK-based compositions. Considerably less attention has been paid to highly filled composites based on other high-performance thermoplastics such as, for example, polyphenylsulfone (PPSU) and polyetherimide (PEI), although these polymers combine high glass transition temperatures, radiation and light resistance, thermo-oxidative stability, good mechanical properties, and melt-processability.

Polyphenylsulfone (PPSU), also referred to as poly(biphenyl ether sulfone), is an amorphous or semi-crystalline sulfone-containing thermoplastic, synthesized by nucleophilic aromatic polycondensation of 4,4′-dihydroxybiphenyl (DHBP; 4,4′-biphenol) with 4,4′-dichlorodiphenyl sulfone (DCDPS) [49,50,51]. The biphenyl aromatic sulfone-containing macromolecular backbone (Figure 1a) distinguishes PPSU from conventional polysulfone and provides a high glass transition temperature of approximately 220 °C, while the ether linkages retain chain mobility sufficient for melt processing. Reported mechanical characteristics of unfilled PPSU include tensile strength of about 60–90 MPa, tensile modulus of about 2.0–2.5 GPa, high elongation before fracture, and high impact strength [51,52,53]. PPSU is also processable from the viscous melt. Commercial grades are processed by injection molding and extrusion at temperatures in the range of approximately 340–390 °C, and PPSU has been specifically evaluated as a high-performance thermoplastic material for large-format additive manufacturing [54]. Furthermore, PPSU can be filled with high contents of particulate fillers without complete loss of melt flow, which is important for melt-based processing routes [55]. This property profile makes PPSU a promising high-performance thermoplastic binder for highly filled LR simulant composites.

Polyetherimide (PEI) is an amorphous aromatic thermoplastic typically obtained from bisphenol A dianhydride (BPADA) and m-phenylenediamine, which form a rigid backbone containing imide rings and ether linkages (Figure 1b). Commercial PEI grades, such as ULTEM™ resins (SABIC, Saudi Arabia), exhibit a T_g_ ≈ 217 °C, tensile strength of approximately 80–100 MPa, and tensile modulus of about 3.2–3.4 GPa, while remaining processable from the melt, with typical injection-molding and extrusion melt temperatures of approximately 350–380 °C [56,57,58,59]. For space-related applications, PEI is particularly relevant. Due to their low outgassing, low smoke generation, reduced flammability, high heat resistance, dimensional stability, melt processability, and retention of mechanical performance at elevated temperatures, PEI-based materials have been actively investigated for space-oriented applications and have been considered and recommended for specific space-related uses in NASA studies [60,61,62,63]. Furthermore, the operating temperature range of PEI-based materials can be extended by chemical modification of the macromolecular structure through the use of alternative diamine and dianhydride components [64,65]. For example, ULTEM™ XH6050, a modified PEI based on BPADA and 4,4′-diaminodiphenyl sulfone (DDS), exhibits T_g_ ≈ 247–248 °C, which is about 30 °C higher than that of standard ULTEM 1000-type PEI grades [56]. This increase in T_g_ makes such modified PEI materials more suitable for thermally demanding lunar surface application scenarios.

Thus, LR/thermoplastic composites represent a promising class of materials for lunar construction, combining high local-resource content with comparatively low-temperature processing conditions. However, despite the growing number of studies on LR simulant/thermoplastic systems, highly filled composites based on high-performance thermoplastics remain insufficiently studied. Therefore, the aim of this work is to develop highly filled LR simulant composites based on PPSU and modified PEI with enhanced T_g_ and to study their structural, mechanical, thermal, and thermomechanical properties as candidate materials for lunar construction applications. The novelty of this work lies in the systematic investigation of these PPSU- and PEI-based systems over a broad filler-content range, including compositions with very high LR contents. The obtained results expand the range of high-performance thermoplastics considered for LR-based construction materials and clarify the composition-dependent balance between local-resource content, consolidation quality, mechanical performance, dimensional stability, and processability of such materials.

## 2. Materials and Methods

### 2.1. Materials

#### 2.1.1. Lunar Regolith Simulant

The LR simulant grade VI-75, developed by GEOKHI RAS (Moscow, Russia) as an engineering analog of lunar soil reproducing the main engineering characteristics of lunar soil relevant to construction studies, including particle-size distribution, particle morphology, density, and physical and mechanical properties, was used as the mineral filler in this study [66,67]. It is a multicomponent mineral powder material prepared from coal-combustion ash, metallurgical slag, and crushed quartz sand, with an average particle density of 2.56 g/cm^3^ (Figure 2). These constituents form a predominantly inorganic silicate-rich material, similar to LR in terms of its general chemical nature (Table 1). Thus, VI-75 can be considered a fine, mineral lunar soil analog suitable for physical and mechanical simulation of lunar regolith in construction-related experiments.

Prior to composite fabrication, the simulant was sieved through a 100 µm sieve, and the <100 µm fraction was used to promote a more uniform filler distribution within the polymer matrix, improve compositional homogeneity, and minimize spatial variations in composite properties.

#### 2.1.2. PPSU and PEI Synthesis

PPSU was synthesized according to the method described in [68,69]. Briefly, DHBP (Hangzhou Hailan Chemical Co., Ltd., Hangzhou, China, 99.5%) and DCDPS (Hangzhou Hailan Chemical Co., Ltd., Hangzhou, China, 99.5%) were dissolved in N,N-dimethylacetamide (DMAA, EKOS-1, Moscow, Russia, 99%), and nucleophilic aromatic polycondensation was carried out in the presence of potassium carbonate (RusChem, Moscow, Russia, 99.5%) as a base at 110–165 °C for 24 h, with removal of water formed during the reaction. The resulting polymer was precipitated into distilled water and washed six times with hot distilled water to remove residual solvent and inorganic salts. The synthesis scheme of PPSU is shown in Figure 3.

PEI was synthesized by a two-step procedure described in [70]. Briefly, in the first stage, a polyamic acid (PAA) solution was prepared at room temperature in N-methyl-2-pyrrolidone (NMP, EKOS-1, Moscow, Russia, 99%) via the reaction of BPADA (Hebei Guanlang Biotechnology Co., Shijiazhuang, China, 99.5%) with DDS (Hebei Guanlang Biotechnology Co., Shijiazhuang, China, 99.5%). In the second stage, thermal imidization of PAA was carried out in solution at 150–180 °C for 6–12 h using pyridine (Py, EKOS-1, Moscow, Russia, 99%) as a catalyst. The progress of the cyclization reaction was monitored by FTIR by recording the gradual decrease and disappearance of the absorption band at 1665 cm^−1^, characteristic of the amide group in the PAA structure, along with the increasing intensity of the bands with maxima at 1720 and 1780 cm^−1^, which are characteristic of the imide ring. The resulting polymer was precipitated into distilled water and washed six times with hot distilled water to remove residual solvent. The synthesis scheme of PEI is shown in Figure 4.

### 2.2. Lunar Regolith Simulant Characterization

#### 2.2.1. Particle-Size Distribution

The particle-size distribution of the LR simulant was determined by dry sieving using a PE-6700 electrodynamic vibration shaker (Ekros Ecohim, Moscow, Russia) fitted with a stack of analytical sieves with different aperture sizes.

#### 2.2.2. Laser Scanning Confocal Microscopy

The morphology of the VI-75 LR simulant particles was examined using an Olympus LEXT OLS5100 3D measuring laser microscope (Olympus, Tokyo, Japan). A small amount of dry powder was placed on the microscope stage and analyzed without additional surface preparation or conductive coating. The particles were imaged at different magnifications using the color imaging and laser scanning modes of the instrument. Low-magnification images were used to evaluate the general particle-size heterogeneity of the powder, while higher-magnification images were acquired using a 100× LEXT objective in 3D Standard + Color mode to examine particle shape, surface relief, and local morphological features.

### 2.3. PPSU and PEI Characterization

#### 2.3.1. FTIR

The chemical structure of the synthesized polymers was confirmed by Fourier-transform infrared spectroscopy (FTIR) using a Nicolet iS10 spectrophotometer (Thermo Scientific, Madison, WI, USA) in attenuated total reflectance (ATR) mode with a germanium prism over the spectral range of 4000–650 cm^−1^.

#### 2.3.2. GPC

The number-average (M_n_) and weight-average (M_w_) molecular weights of the synthesized PPSU and PEI samples were determined by gel permeation chromatography (GPC) using a Shimadzu Prominence LC-20 system (Shimadzu, Kyoto, Japan) equipped with an RID-20A refractive index detector and an SPD-20A UV detector. Three PSS GRAM connected in series analytical columns (PSS Polymer, Mainz, Germany) with pore sizes ranging from 100 to 3000 Å were used. N,N-dimethylformamide (DMF, EKROS, Moscow, Russia, 99%) containing 0.01 M lithium bromide (99.9%, Ruskhim, Moscow, Russia) was used as the eluent at a flow rate of 1.0 mL/min and a column temperature of 40 °C. Calibration was performed using a set of polymethyl methacrylate standards (PSS Polymer, Mainz, Germany) with molecular weights ranging from 0.8 to 1200 kDa.

#### 2.3.3. DSC

The glass transition temperature (T_g_) of the synthesized PPSU and PEI was determined by differential scanning calorimetry (DSC) using a NETZSCH DSC 204 F1 Phoenix instrument (NETZSCH, Selb, Germany) under an argon atmosphere at a heating rate of 10 °C/min over the temperature range of 25–400 °C.

#### 2.3.4. TGA

The thermo-oxidative stability of melts of the synthesized PPSU and PEI binders, quantified as the temperature corresponding to 5% mass loss (T_5%_), was evaluated by thermogravimetric analysis (TGA) using a NETZSCH TG 209 F1 Libra instrument (NETZSCH, Selb, Germany) over the temperature range of 20–700 °C at a heating rate of 10 °C/min in an air atmosphere.

### 2.4. Composite Fabrication

After synthesis, washing, and drying, the PPSU and PEI binders were mechanically ground using a high-speed knife mill. The resulting polymer powders were sieved to obtain a fraction with particle sizes below 100 µm.

LR simulant/high-temperature thermoplastic composites were prepared using a powder-processing route based on dry blending followed by hot pressing. The compositions of the LR simulant/thermoplastic composites, with LR simulant contents ranging from 50 to 92.5 wt.%, were selected to minimize the polymer content while enabling the production of hot-pressed specimens with sufficient strength for further investigation. For each system, the regolith simulant and polymer powder were weighed according to the target mass ratios and placed into a laboratory M-5L 3D Tumbler Mixer (ZyLAB Technologies, Amsterdam, The Netherlands). Dry mixing was carried out for 30–80 min until a visually homogeneous powder blend was obtained.

The prepared powder blends were consolidated by hot pressing in a closed cylindrical steel mold with an inner diameter of 70 mm. The required amount of the powder blend (~28–30 g), sufficient to produce a disc approximately 4–6 mm thick, was loaded into the mold cavity and placed between the heated platens of a hydraulic hot press. The pressing temperature was selected according to the binder type. PPSU-based composites were pressed at 330 °C, whereas PEI-based composites were pressed at 340 °C. During heating, the pressure was increased stepwise from 1 to 5 MPa to ensure gradual compaction of the powder mixture, improved wetting of regolith particles by the molten polymer binder, and removal of air. The final pressure of 5 MPa was maintained during the isothermal holding stage at the target temperature for 30 min to ensure complete consolidation of the composite structure. After holding, heating was stopped, and the mold was cooled under pressure. The samples were demolded when the mold temperature decreased to approximately 100 °C and were then cooled to room temperature in air.

### 2.5. Composite Investigation

#### 2.5.1. Density Measurement

The geometric density of the hot-pressed composite disks was determined by the geometric method from direct measurements of mass and dimensions of the specimens. The mass of each specimen was measured using an analytical balance, while the disk diameter and thickness were measured using a digital caliper and micrometer (Finch Manufacturing & Technology, West Pittston, PA, USA), respectively. The measurements were taken at several points, including the center and peripheral regions of the disk, and the average value was used for volume calculation.

#### 2.5.2. X-Ray Micro-Computed Tomography

The internal pore structure of the hot-pressed composite specimens was analyzed by X-ray micro-computed tomography (micro-CT) using a Bruker SkyScan system (Bruker, Kontich, Belgium). The specimens were scanned at an accelerating voltage of 50 kV with a rotation step of 0.4°. The voxel size was 5 µm, and virtual cross-sections were reconstructed with a slice spacing of 5 µm. No image smoothing was applied during reconstruction. The reconstructed image stacks were processed using CTAn software (version 1.20.8, Bruker, Kontich, Belgium). Porosity was determined by grayscale threshold segmentation, where the pore phase was identified as the dark regions within the reconstructed volume. The total porosity was calculated as the volume fraction of segmented pores relative to the analyzed specimen volume. Two-dimensional X-ray projection images acquired during micro-CT scanning were additionally used for a qualitative assessment of large-scale density heterogeneity and possible filler segregation in the composite specimens.

#### 2.5.3. Shore D Hardness

The indentation hardness of the hot-pressed composite disks was determined on the Shore D scale in accordance with [71]. Measurements were performed using a Bareiss BS 61 II Shore hardness tester (Bareiss Prüfgerätebau GmbH, Oberdischingen, Baden-Württemberg, Germany). For each composition, four measurements were taken at different points on the disk surface, and the average value was reported.

#### 2.5.4. Compression Tests

The mechanical properties of the LR simulant/thermoplastic composites under compressive loading were determined in accordance with [72] using a Zwick/Roell universal testing machine (ZwickRoell, Ulm, Germany). Rectangular specimens of approximately 15 mm × 5 mm × 4 mm nominal dimensions were cut from the hot-pressed composite disks. The tests were performed at a crosshead speed of 1 mm/min. Load–displacement curves were recorded and converted into stress–strain curves using the initial specimen dimensions. The compressive strength (σ_c_) was determined as the maximum compressive stress reached during the test. For each composition, three specimens were tested, and the mean value was calculated from the obtained results. The compressive modulus (E_c_) was calculated from the initial linear region of the stress–strain curve.

#### 2.5.5. Thermal Conductivity

The thermal conductivity tests of the hot-pressed composite disks were performed with an ITP-MG4 thermal conductivity meter (Stroypribor, Chelyabinsk, Russia). During the test, the disk specimen was placed between heated and cooled plates, and a stationary heat flux was passed through the specimen perpendicular to its flat surfaces. After reaching steady-state conditions, the thermal conductivity value was calculated automatically by the instrument based on the measured heat flow, temperature difference, and specimen thickness.

#### 2.5.6. CLTE

The coefficient of linear thermal expansion (CLTE) of the composite specimens was determined by dilatometry using a Linseis DIL L75 VS vertical push-rod dilatometer (Linseis Messgeräte GmbH, Selb, Germany). Rectangular specimens cut from the hot-pressed disks were heated from 20 to 150 °C, and the relative length change was recorded as a function of temperature. To minimize the influence of residual processing stresses and adsorbed moisture, the CLTE values were determined from the second heating run. The average CLTE was calculated over the selected temperature interval.

#### 2.5.7. DMA

The thermomechanical behavior of the LR simulant/thermoplastic composites was evaluated by dynamic mechanical analysis (DMA) using a NETZSCH DMA 242 E Artemis instrument (NETZSCH, Selb, Germany). Rectangular specimens were cut from the hot-pressed composite disks and tested in a three-point bending configuration. The measurements were performed over the temperature range of 20–270 °C at a heating rate of 2 °C/min and a frequency of 1 Hz. During the test, the dynamic deformation response of the specimens under oscillatory loading was recorded, and the storage modulus (E′) and loss tangent (tanδ) were calculated from the amplitude and phase shift of the mechanical response.

#### 2.5.8. Melt Flow Index

The Melt Flow Index (MFI) of the PPSU and PEI binders, as well as their corresponding LR simulant/thermoplastic composites, was determined using a PTR-LAB-11 device (LOIP, Saint Petersburg, Russia) according to [73] under a load of 5 kg. The results were expressed in g/10 min.

#### 2.5.9. Graphical Abstract Preparation

Selected visual elements of the graphical abstract were generated using ChatGPT (ChatGPT Images 2.5, OpenAI, San Francisco, CA, USA) and subsequently reviewed and incorporated into the final composition by the authors using Adobe Photoshop (version 27.10, Adobe Inc., San Jose, CA, USA).

## 3. Results

### 3.1. Morphology and Particle-Size Distribution of the VI-75 Lunar Regolith Simulant

The particle-size distribution of the VI-75 LR simulant is presented in Table 2. The simulant was characterized by a predominantly fine particle-size distribution. The main fraction corresponded to particles in the range of 0.05–0.01 mm and accounted for 49.8 wt.%, while the 0.1–0.05 mm fraction contributed 39.0 wt.%. Thus, particles within the 0.1–0.01 mm range represented the major part of the material. Smaller particles below 0.01 mm accounted for 10.9 wt.% in total.

The micrographs of the VI-75 LR simulant are shown in Figure 5. Reflected-light optical microscopy at 20× magnification revealed a heterogeneous powder consisting of particles with different sizes and colors. Higher-magnification laser scanning confocal microscopy confirmed the irregular morphology of particles of regolith simulant. The particles exhibited angular and subangular shapes, rough surfaces, and a broad size distribution, which is typical for crushed mineral powders used as LR simulants.

### 3.2. Characterization of PPSU and PEI Binders

The synthesized PPSU and PEI binders were characterized by FTIR (Figure S1), GPC (Figure S2), DSC (Figure S3), TGA (Figure S4), and MFI measurement. The molecular weight characteristics, glass transition temperatures, melt thermal stability, and melt flow indices of the binders are summarized in Table 3.

### 3.3. Composite Samples

The resulting composites containing 50–90 wt.% of LR simulant for PPSU-based systems and 50–92.5 wt.% of LR simulant for PEI-based systems were obtained as circular disks with a diameter of 70 mm and a thickness of approximately 4–5 mm (Figure 6). PPSU-based composites containing 92.5 wt.% of LR simulant and PEI-based composites with LR simulant contents above 92.5 wt.% did not possess sufficient mechanical integrity and disintegrated during demolding. The calculated LR simulant volume fraction was determined from the target mass composition using the particle density of VI-75 and the density of the corresponding neat polymer (Table 4).

A two-dimensional X-ray projection image of the hot-pressed LR simulant/PEI composite specimen containing 50 wt.% of the filler is shown in Figure 7. The darker inclusions are attributed to the LR simulant filler phase, which has higher X-ray attenuation than the polymer binder. These inclusions were distributed across the projected area of the specimen, and no large particle-accumulation zones or visually distinguishable polymer-rich regions were observed at the examined scale.

### 3.4. Density and Porosity

The geometric density and porosity of the hot-pressed LR simulant/thermoplastic composites were evaluated from the specimen mass and geometric volume and by micro-CT analysis, respectively. Representative reconstructed micro-CT images of the LR simulant/PEI composites containing 50 and 80 wt.% of the filler are shown in Figure 8.

In the grayscale reconstructions, regions with higher intensity, corresponding to stronger X-ray attenuation, appear as light gray to white and correspond predominantly to the denser mineral filler, whereas regions of lower attenuation appear darker and are associated with the less dense polymer matrix; the darkest regions correspond to pores. Comparison of Figure 8a,b showed that the composite containing 80 wt.% of LR simulant had a substantially greater proportion of high-attenuation filler regions than the 50 wt.% composite, while the matrix-rich regions became correspondingly less widespread. In both composites, filler-associated regions were distributed throughout the analyzed cross-section, and no large continuous filler-rich or matrix-rich regions indicative of pronounced macroscopic phase segregation were observed. At the same time, local variations in the relative proportions of the filler and matrix phases are apparent. Isolated pores are observed in both specimens, with the pore regions being more pronounced in the composite containing 80 wt.% of the filler.

The false-color rendering of the 80 wt.% composite provides an enhanced visualization of the attenuation contrast observed in the corresponding grayscale reconstruction (Figure 8c). In this representation, the higher-attenuation regions associated predominantly with the mineral filler are displayed mainly in yellow to white, whereas the lower-attenuation polymer matrix is represented predominantly by pink/purple regions; the darkest regions correspond to pores. Intermediate colors at the interfaces between these regions reflect intermediate reconstructed attenuation values and cannot be assigned unambiguously to an individual phase. The enlarged region in Figure 8d illustrates representative filler, matrix, and pore regions and facilitates comparison of their characteristic attenuation levels.

It should be emphasized that, at the available spatial resolution, the micro-CT images primarily provide a qualitative assessment of the internal structure and macrohomogeneity of the composites; individual fine LR simulant particles, thin matrix regions, and their interfaces cannot be fully resolved, and partial-volume effects can result in overlapping attenuation ranges between adjacent phases. Consequently, the reconstructed images demonstrate the overall distribution of filler- and matrix-rich regions and the presence of pores, but do not permit precise reconstruction of the local filler distribution or unequivocal segmentation of all individual filler particles, matrix regions, and pores.

The relationships between geometric density and porosity with LR simulant content are shown in Figure 9. The systems based on PPSU and PEI exhibited different density and porosity trends. For PPSU-based composites, the geometric density was highest at 50 wt.% of LR simulant and continuously decreased from 1.72 to 1.44 g/cm^3^ as the filler content increased to 90 wt.%. This change was accompanied by a continuous increase in porosity over the investigated composition range. In contrast, the PEI-based composites showed a continuous increase in geometric density from 1.64 g/cm^3^ at 50 wt.% of LR simulant to 2.04 g/cm^3^ at 92.5 wt.%, although the increase became less pronounced at the highest filler loadings. The porosity of the PEI-based composites remained comparatively low (~5–6 vol.%) at 50–70 wt.% of LR simulant, but increased more noticeably at 85 wt.%, and reached approximately 13 vol.% at 90–92.5 wt.%.

In LR-based materials, the level of residual porosity is closely related to the combination of properties of the material. For load-bearing applications, lower porosity is generally favorable because increasing pore content reduces the effective load-bearing cross-section and facilitates stress concentration, resulting in lower mechanical performance [48,74]. At the same time, porosity also affects thermal conductivity, dimensional stability, dielectric behavior, gas permeability, radiation-shielding performance, etc. Consequently, the acceptable porosity level should be considered in relation to the specific functional requirements of the material and may therefore be deliberately adjusted.

### 3.5. Mechanical Properties

#### 3.5.1. Shore D Hardness

The Shore D hardness of the LR simulant/thermoplastic composites as a function of filler content is shown in Figure 10. For both binder systems, the incorporation of the LR simulant increased the surface hardness relative to the corresponding neat polymer, from 75 to 88 for PPSU-based composites and from 86 to 96 for PEI-based composites. For PPSU-based composites, the hardness remained nearly unchanged between 50 and 60 wt.% of the filler and then gradually decreased with further increase in LR simulant content. A similar trend was observed for PEI-based composites, although this system exhibited higher hardness values over the entire investigated composition range. At the highest filler loadings, the Shore D hardness of the PEI-based composites decreased more noticeably to 81 at 92.5 wt.% of the filler.

#### 3.5.2. Compression Tests

The dependence of compressive strength (σ_c_) and compressive modulus (E_c_) on LR simulant content is shown in Figure 11 and Tables S1 and S2. The two systems exhibited similar compressive behavior over the investigated composition range. For PPSU-based composites, σ_c_ decreased from 93 MPa for neat PPSU to 80 MPa at 50 wt.%, then increased with filler content up to 70 wt.%, reaching 122 MPa, and then sharply decreased at higher LR simulant loadings; E_c_ followed a generally similar trend, reaching its highest value of 6.3 GPa at 80 wt.% of the filler, followed by a dramatic decrease.

For PEI-based composites, the σ_c_ increased from 150 MPa for neat PEI to a maximum of 193 MPa at 60 wt.% of the filler. With further increase in LR simulant content, σ_c_ sharply decreased, reaching 13 MPa at 92.5 wt.% of the filler. E_c_ showed the same general trend, with the highest value of 7.4 GPa observed at 60 wt.% of the filler, followed by a progressive decrease at higher filler contents.

### 3.6. Thermal Properties

The thermal properties of the LR simulant/thermoplastic composites were evaluated in terms of thermal conductivity and linear thermal expansion; the obtained data are shown in Figure 12.

#### 3.6.1. Thermal Conductivity

For both LR simulant/thermoplastic systems, λ increased with increasing filler content over the investigated composition range. In the PPSU-based composites, the thermal conductivity increased from 0.35 W/(m·K) for the neat polymer to 0.64 W/(m·K) at 90 wt.% of the filler. A similar trend was observed for the PEI-based composites, where λ increased from 0.22 W/(m·K) for neat PEI to 0.72 W/(m·K) at 92.5 wt.% of the filler. At high filler loadings, the PEI-based composites had slightly higher λ values, which is consistent with their lower porosity.

#### 3.6.2. CLTE

In contrast to thermal conductivity, the CLTE decreased steadily with increasing filler content for both composite systems. For the LR simulant/PPSU composites, the CLTE decreased from 61 × 10^−6^ K^−1^ for the neat polymer to 14.2 × 10^−6^ K^−1^ at 90 wt.% of the filler. The PEI-based composites showed a similar dependence; CLTE decreased from 52 × 10^−6^ K^−1^ for neat PEI to 10.1 × 10^−6^ K^−1^ at 92.5 wt.% of the filler. Across comparable compositions, the PEI-based composites generally exhibited lower CLTE values than the PPSU-based ones.

### 3.7. Thermomechanical Transition Temperatures

DMA was performed for the neat polymers and for the LR simulant/thermoplastic composites (Figures S5 and S6). For both PPSU- and PEI-based systems, DMA specimens were successfully obtained for compositions containing 0, 50, 60, 70, and 80 wt.% of LR simulant. At higher LR simulant contents, the specimens were characterized by pronounced brittleness and fractured during cutting from the hot-pressed disks; therefore, they could not be tested by DMA.

The dependence of the characteristic transition temperatures determined from storage modulus E′ and loss tangent tanδ on LR simulant content is shown in Figure 13. For PPSU-based composites, T_g_(E′) remained within a relatively narrow range from 196.7 to 207.0 °C over the investigated composition range. T_g_(tanδ) showed a gradual increase with increasing LR simulant content, rising from 210.4 °C for neat PPSU to 226.9 °C at 80 wt.% of the filler. For PEI-based composites, T_g_(E′) decreased from 219.4 °C for neat PEI to 201.5 °C at 50 wt.% of the filler and then increased with further increase in LR simulant content, reaching 222.6 °C at 70 wt.% of the filler and remaining at a similar level at 80 wt.% of the filler. T_g_(tanδ) showed the same general trend: it decreased from 248.2 °C for neat PEI to 216.7 °C at 50 wt.% of the filler and then gradually increased to 243.3 °C at 80 wt.% of the filler. The T_g_ values obtained for neat PPSU and DDS-based PEI by DMA were in good agreement with the corresponding values reported in the literature for commercial grades of these polymers [75,76,77].

### 3.8. Melt Flow Index

The MFI decreased markedly after incorporation of the LR simulant for both binder systems (Figure 14). However, the composites containing up to 60 wt.% of the filler retained melt/flow capability under the selected test conditions (340 °C for PPSU and 370 °C for PEI). For PPSU-based composites, the MFI decreased from 18.1 g/10 min for neat PPSU to 4.7 and 1.8 g/10 min at 50 and 60 wt.% of the filler, respectively. For PEI-based composites, the MFI decreased from 32.0 g/10 min for neat PEI to 8.5 and 7.8 g/10 min at 50 and 60 wt.% of the filler, respectively. At higher LR simulant contents, no measurable melt flow was detected under the test conditions.

## 4. Discussion

### 4.1. Binder and Lunar Regolith Simulant Characterization

Based on laser scanning confocal microscopy (Figure 5) and particle-size analysis (Table 2), LR simulant VI-75 can therefore be regarded as a polydisperse, angular mineral filler with a predominantly fine particle-size distribution. The fact that the size of 88.8 wt.% of the particles were within the 0.1–0.01 mm range indicates that VI-75 can provide a large filler–binder interfacial area in the composite. Therefore, the properties of LR simulant/thermoplastic composites can be strongly affected by particle distribution within the polymer matrix, binder wetting of the mineral phase, pore formation, possible particle agglomeration, and other interfacial effects.

The chemical structures of the synthesized polymers were confirmed by FTIR spectroscopy; the characteristic absorption bands were consistent with the corresponding chemical groups of PPSU and PEI and literature data (Figure S1) [78,79]. The polymer peaks on GPC chromatograms of both PPSU and PEI corresponded to unimodal molecular-weight distributions (Figure S2, Table 3). The DSC thermograms of PPSU and PEI exhibited a single endothermic step corresponding to the glass transition, with T_g_ of 219.1 °C and 247.1 °C, respectively (Figure S3, Table 3). No exothermic crystallization peaks or endothermic melting peaks were observed, confirming the amorphous nature of both polymers. The higher T_g_ of PEI can be attributed to the presence of polar imide groups, which enhance intermolecular interactions and restrict segmental mobility of the polymer chains compared with PPSU [65,70]. Both binders exhibited high heat resistance and thermo-oxidative stability: T_g_ values were 219.1 °C for PPSU and 247.1 °C for PEI, while T_5%_ values reached 483.1 and 510.2 °C, respectively (Table 3). These characteristics confirm that the selected high-temperature thermoplastic polymers can be considered as promising binders for composites intended for operation under the thermally demanding conditions of the lunar surface. The synthesized PPSU and PEI also had comparable molecular-weight characteristics, with M_w_/M_n_ values of 51.4/24.2 kDa and 55.3/21.3 kDa, respectively. At the same time, both polymers retained good melt/flow capability, with MFI values above 15 g/10 min under the selected test conditions (5 kg load and 340 and 370 °C for PPSU and PEI, respectively), which makes both of these binders and filled composites based on them potentially suitable for processing by additive manufacturing technologies.

### 4.2. Internal Structure of the Hot-Pressed Lunar Regolith Simulant/Thermoplastic Composites

Hot pressing of powder mixtures made it possible to obtain disk-shaped LR simulant/thermoplastic composites over a wide filler-content range of 50–92.5 wt.%. The X-ray projection image of the composite containing 50 wt.% of the filler showed that the mineral phase was distributed throughout the specimen without clearly visible large filler- or polymer-rich zones (Figure 6), which indicates that the selected dry blending and hot-pressing route provided macroscopic-scale uniform distribution of the LR simulant in the polymer matrix. Reconstructed micro-CT images further confirmed the heterogeneous internal structure typical of particulate composites (Figure 8). The presence of isolated low-attenuation regions indicated that pore formation was not fully eliminated even at 50 wt.% of the filler. Therefore, the internal structure of the composites should be considered as a three-phase system consisting of polymer binder, mineral filler, and residual porosity.

The porosity generally increased with increasing LR simulant content, which can be attributed to the decreasing amount of polymer binder available for wetting, filling the interparticle space, and consolidating the mineral phase. For PPSU-based composites, porosity increased from 4.31% at 50 wt.% of the filler to 38.76% at 90 wt.%. In contrast, PEI-based composites retained considerably lower porosity, remaining within 5.08–5.58% up to 80 wt.% of the filler and increasing to only about 13.3% at 90–92.5 wt.%. This difference may indicate more efficient wetting of the LR simulant by the PEI matrix and more effective filling of voids between mineral particles during hot pressing. This structural difference was also reflected in the visual appearance of the hot-pressed disks (Figure 6). Composites with a higher polymer-binder content appeared darker, whereas highly filled composites with a lower polymer content became gray, which may be attributed to enhanced light scattering by the more porous, mineral-rich structure.

### 4.3. Properties of Lunar Regolith Simulant/Thermoplastic Composites

#### 4.3.1. Density

The geometric density of the composites was governed by two opposite factors: the increasing content of the mineral filler, which has a higher intrinsic density than the polymer binder, and the formation of residual porosity during hot pressing. For PPSU-based composites, the density increased from 1.29 g/cm^3^ for neat PPSU to 1.72 g/cm^3^ at 50 wt.% of LR simulant and subsequently decreased to 1.44 g/cm^3^ at 90 wt.%. For PEI-based composites, the density increased from 1.31 g/cm^3^ for neat PEI to 1.64 g/cm^3^ at 50 wt.% of LR simulant content and then progressively increased to 2.04 g/cm^3^ at 92.5 wt.% (Figure 9). This behavior is consistent with the increase in porosity in PPSU-based composites discussed above and indicates that, for this binder system, the effect of pore formation at high filler contents outweighed the density increase expected from the higher fraction of the mineral phase. By contrast, the PEI-based composites retained substantially lower porosity and exhibited a continuous increase in geometric density over the investigated composition range, indicating more effective consolidation than the PPSU-based system under the selected hot-pressing conditions.

#### 4.3.2. Mechanical Properties

Shore D hardness, compressive strength, and compressive modulus exhibited non-monotonic dependences on LR simulant content, with maxima in the range of 50–80 wt.% of the filler (Figure 10 and Figure 11). For both systems, the highest surface hardness was achieved at 50–60 wt.% of LR simulant content: Shore D hardness reached 88 and 96 for PPSU- and PEI-based composites, respectively. In compression tests, PPSU-based composites reached a maximum σ_c_ of 122 MPa at 70 wt.% of LR simulant and a maximum E_c_ of 6.3 GPa at 80 wt.%, whereas the PEI-based composite containing 60 wt.% of LR simulant exhibited the highest overall strength and stiffness, with σ_c_ = 193 MPa and E_c_ = 7.4 GPa. With further increases in LR simulant content beyond the maxima, the compressive strength of LR simulant/thermoplastic composites progressively decreased, ultimately falling below 20 MPa at LR simulant contents of 90 wt.% for PPSU-based composites and 92.5 wt.% for PEI-based composites.

This behavior reflects the competition between mineral-particle reinforcement and the deterioration of composite integrity at high filler contents. At lower and intermediate LR simulant content, the rigid particles restrict deformation of the polymer matrix and improve stress transfer within the sample. However, at excessive filler contents, the polymer-binder amount becomes insufficient for complete consolidation of the mineral phase, resulting in poorer matrix continuity and higher residual porosity (Figure 9). Therefore, after reaching the maxima, the reinforcing effect of the LR simulant is outweighed by structural defects, ultimately leading to brittle behavior and a sharp decrease in load-bearing capacity. Other microstructural factors may also contribute to the deterioration of mechanical properties at high LR simulant contents. The angular morphology of the particles may hinder dense packing and promote local stress concentrations (Figure 5); increasing the surface of contact between LR particles and their possible local agglomeration may reduce the continuity of the polymer phase, separating adjacent particles and creating regions of structural heterogeneity. However, the individual contribution of these factors could not be resolved in the present study because the spatial resolution of the applied imaging methods was insufficient for detailed analysis of local particle contacts and small-scale heterogeneity.

Importantly, despite the pronounced decrease in mechanical properties at high LR simulant contents, the LR simulant/thermoplastic composites retained compressive strengths of approximately 50 and 30 MPa and compressive moduli of 6.3 and 3.2 GPa at LR simulant contents of 80 and 90 wt.% for the PPSU- and PEI-based systems, respectively. These values substantially exceed the compressive strengths of many types of clay bricks used in load-bearing masonry and are comparable to those of normal-strength structural concretes used in terrestrial construction, including multistorey buildings [80,81]. Moreover, the gravitational acceleration at the lunar surface is 1.625 m/s^2^, approximately one-sixth of that on Earth [82]; therefore, stresses arising from the self-weight of a structure under lunar gravity are approximately six times lower than on Earth. Accordingly, the combination of retained compressive strength, reduced gravitational loading, and high LR simulant content (up to 80 wt.% for PPSU and up to 90 wt.% for PEI) supports the potential use of these composites for hot-pressed construction blocks and other compression-dominated structural elements on the lunar surface.

Comparison with previously reported LR/polymer systems (Table 5) shows that the developed PEI- and PPSU-based composites exhibit competitive compressive performance among LR-filled thermoplastics. In particular, PEI-based composites demonstrate strengths comparable to or higher than those reported for PLA, PE, PEEK, and PAEK systems over a broad range of LR contents. A larger body of literature is available for thermosetting epoxy-based LR composites. Depending on resin chemistry, filler content, processing route, and other factors, their reported compressive strengths vary widely, from approximately 10 MPa to above 100 MPa. At intermediate LR contents of up to approximately 80–85 wt.%, the PEI-based composites developed in the present study exhibited compressive strengths comparable to or higher than those reported for epoxy-based systems (Table 5). However, published data show that a number of epoxy-based LR composites retain compressive strengths above 20–30 MPa even at substantially higher LR contents of up to 97–98 wt.%. This behavior at extremely high filler loadings may be associated with the relatively low viscosity of uncured resin systems, which facilitates binder distribution, wetting of the regolith particles, and filling of interparticle voids, thereby limiting residual porosity. Nevertheless, high-performance thermoplastics offer a different set of advantages, including generally higher heat resistance and thermo-oxidative stability than conventional epoxy matrices, as well as the potential for repeated melt processing, repair, and recycling. These features may be particularly relevant to long-term lunar manufacturing scenarios, where material reuse and repair could reduce dependence on supplies transported from Earth. Thus, selection of the polymer binder should not be based solely on the mechanical performance of the resulting composite, but should also account for application-specific requirements, including thermal stability, processing conditions, recyclability, binder demand, the intended manufacturing route, etc. It should also be noted that direct quantitative comparison of the reported mechanical properties is inherently limited by substantial differences in the nature of regolith simulants, composite preparation conditions, and mechanical testing procedures among the studies.

#### 4.3.3. Thermal and Thermomechanical Properties

Thermal conductivity and CLTE showed monotonic trends with increasing LR simulant content. The incorporation of the mineral phase increased thermal conductivity up to 0.64 W/(m·K) for PPSU-based composites and 0.72 W/(m·K) for PEI-based composites (Figure 12a), which can be attributed to the growing fraction of the inorganic mineral phase, promoting more efficient heat conduction through the composite than the neat polymer binder. At the same time, the CLTE of the composites decreased from 50–60 × 10^−6^ K^−1^ for neat polymers to 10–15 × 10^−6^ K^−1^ (Figure 12b) due to the restriction of thermally induced polymer-chain mobility by the rigid filler particles and the replacement of a highly expandable polymer phase with a dimensionally more stable mineral component. For lunar construction applications, this combination of thermal properties is beneficial because the moderate increase in thermal conductivity can reduce local temperature gradients within structural elements, while the simultaneous decrease in CLTE limits thermally induced dimensional changes, which can reduce thermal stresses and help preserve the dimensional integrity of structural elements during repeated thermal cycling. Therefore, the addition of LR simulant potentially improves the ability of the composites to tolerate thermal cycling under the thermal conditions characteristic of the lunar surface.

DMA revealed different patterns of thermomechanical behavior for the two binder systems (Figure 13). For PPSU-based composites, T_g_(tanδ) increased with increasing LR simulant content, from 210.4 °C for the neat polymer to 226.9 °C at 80 wt.% of the filler, while T_g_(E′) remained within a relatively narrow temperature range. In contrast, PEI-based composites showed a non-monotonic dependence: both T_g_(E′) and T_g_(tanδ) decreased after the introduction of 50 wt.% of the filler and then increased again at higher LR simulant contents. The upward shift of T_g_(tanδ) in the PPSU-based system can be associated with restricted segmental mobility of polymer chains near the mineral surface. However, the non-monotonic behavior of PEI-based composites suggests a more complex balance between polymer–filler interactions, matrix continuity, and residual stresses formed during hot pressing. Nevertheless, the characteristic transition temperatures of the tested composites remained above 200 °C, confirming that the high-temperature thermomechanical character of both polymer matrices was retained after filling with LR simulant.

#### 4.3.4. Melt/Flow Behavior

MFI is a key parameter for evaluating the potential applicability of the developed composites in melt-based additive manufacturing on the lunar surface, especially for the fabrication of complex-geometry components. The decrease in MFI with increasing LR simulant content is expected for particulate polymer composites and can be attributed to the increasing solid-particle fraction and restricted mobility of the molten polymer phase. However, the retention of melt/flow capability at 50–60 wt.% of the filler (Figure 14) is crucial because these compositions also showed improved mechanical, thermal, and dimensional characteristics compared with the neat polymers and, most importantly, can reduce the amount of polymer binder that would need to be transported from Earth by more than twofold. Therefore, the 50–60 wt.% composition range can be considered the most promising processing window for future optimization of extrusion-based or other melt-based additive manufacturing approaches for complex-geometry lunar construction components.

### 4.4. Application-Oriented Selection of Composite Compositions

The obtained property set of high-performance LR simulant/thermoplastic composites indicates that, generally, PEI-based systems are more promising than PPSU-based ones for lunar construction applications. Compared with PPSU-based composites, the PEI-based systems showed lower porosity, higher density, compressive strength, and compressive modulus at comparable LR simulant contents. A minor advantage of PPSU-based composites is that they can be processed at lower temperatures, which may modestly reduce energy consumption during fabrication. Therefore, PEI-based composites can be considered as the primary material system, whereas PPSU-based composites may be regarded as an alternative binder system for cases where lower processing temperature is prioritized over maximum mechanical performance.

The selection of the optimal composite composition should depend on the intended application (Table 6). For melt-based additive manufacturing of complex-geometry parts, the most promising compositions are PEI-based composites containing 50–60 wt.% of LR simulant. In this range, the composites retain melt/flow capability, which is essential for extrusion-based shaping, while also showing improved strength, stiffness, hardness, thermal conductivity, and dimensional stability compared with the neat polymer. However, this composition range still requires 40–50 wt.% of polymer binder, which would have to be transported from Earth, and therefore remains the main limitation of their use for lunar construction.

For load-bearing block-type elements of simple geometry that can be produced by hot pressing, the PEI-based composites containing 70–90 wt.% of LR simulant appeared to be the most suitable, since this range provides the best overall balance between high compressive performance, low porosity, improved dimensional stability, and increased utilization of local mineral material, while containing a higher fraction of local LR simulant than the compositions selected for melt-based additive manufacturing. Throughout this composition range, the compressive strength remained at or above 30 MPa, including 30 MPa at an LR simulant content of 90 wt.%. This value is comparable in magnitude to the compressive-strength levels of structural concrete classes used for terrestrial buildings [64], indicating that these highly filled composites retain substantial potential for compression-dominated structural applications. However, this recommendation is based primarily on the compressive performance of the composites and should therefore be regarded as preliminary; the suitability of these composites for structural applications requires further evaluation under tensile and flexural loading and characterization of their fracture behavior.

In contrast, highly filled PEI-based composites containing 92.5 wt.% of LR simulant should be considered as compositions that maximize the fraction of local mineral material and strongly reduce the amount of polymer binder that would need to be transported from Earth. However, their reduced strength, absence of melt flow, and increased brittleness limit their use to simple-shape non-load-bearing or weakly loaded construction elements.

## 5. Conclusions

In this work, LR simulant composites based on high-performance PPSU and modified high-T_g_ PEI thermoplastic binders were prepared by dry blending the LR simulant with the polymer powders, followed by hot pressing. The investigated LR simulant content ranges were 50–90 wt.% for PPSU- and 50–92.5 wt.% for PEI-based composites. At low and intermediate LR contents, mineral filling increased hardness and improved compressive performance, with the maximum compressive strength reaching 122 and 193 MPa and the compressive modulus reaching 6.3 and 7.4 GPa for PPSU- and PEI-based composites, respectively. Increasing LR content also increased thermal conductivity and substantially reduced CLTE, while the high thermomechanical properties for both matrices were retained. However, binder deficiency at excessive filler loadings, corresponding to ≥70 wt.% for PPSU- and ≥85 wt.% for PEI-based composites, resulted in increased porosity and brittleness and decreased compressive strength. Importantly, the melt/flow capability of the composites was retained up to 60 wt.% of LR simulant, indicating the potential for melt-based processing. Among the two investigated binder systems, PEI-based composites demonstrated the most favorable composition–property balance.

The results further demonstrate that the composition of the LR simulant-filled polymer composites should be selected according to the intended application in lunar construction. PEI-based composites containing 50–60 wt.% of LR simulant are promising for melt-based additive manufacturing of complex-geometry parts because they retain melt/flow capability while showing improved mechanical, thermal, and dimensional characteristics compared with the neat polymer binder. PEI-based composites containing 70–90 wt.% of LR simulant retained compressive strength of ≥30 MPa, placing them within or above the strength range relevant to materials used in load-bearing masonry and normal-strength structural concrete applications. Considering that lunar gravity is approximately one-sixth of that on Earth further supports the potential application of these compositions in hot-pressed construction elements. Highly filled PEI-based composites containing 92.5 wt.% of LR simulant maximize the fraction of local mineral material and minimize the amount of polymer binder that would need to be transported from Earth, but their reduced strength, absence of melt flow, and increased brittleness limit their potential use to simple-shape, non-load-bearing, or weakly loaded elements.

Overall, this research demonstrates that LR/thermoplastic composites can be considered promising materials for future lunar construction concepts based on partial utilization of local mineral resources and transported high-performance polymer binders. Further work should focus on extrusion-based processing trials, optimization of printing parameters, and additional testing under conditions relevant to lunar operation, including flexural and tensile loading, fracture behavior, impact, abrasion, thermal cycling, vacuum exposure, and radiation resistance, as well as validation of the obtained trends using LR simulants with different chemical and mineralogical characteristics, including mare- and highland-type simulants.

## Figures and Tables

**Figure 1:**
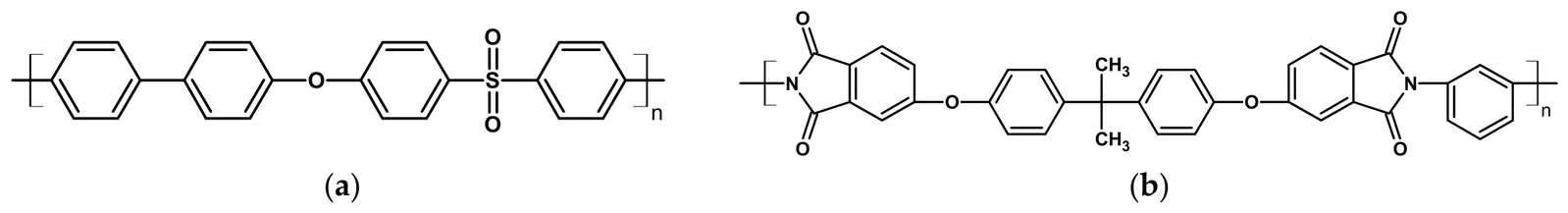
Structural formulas of high-performance thermoplastics: (**a**) polyphenylsulfone; (**b**) polyetherimide.

**Figure 2:**
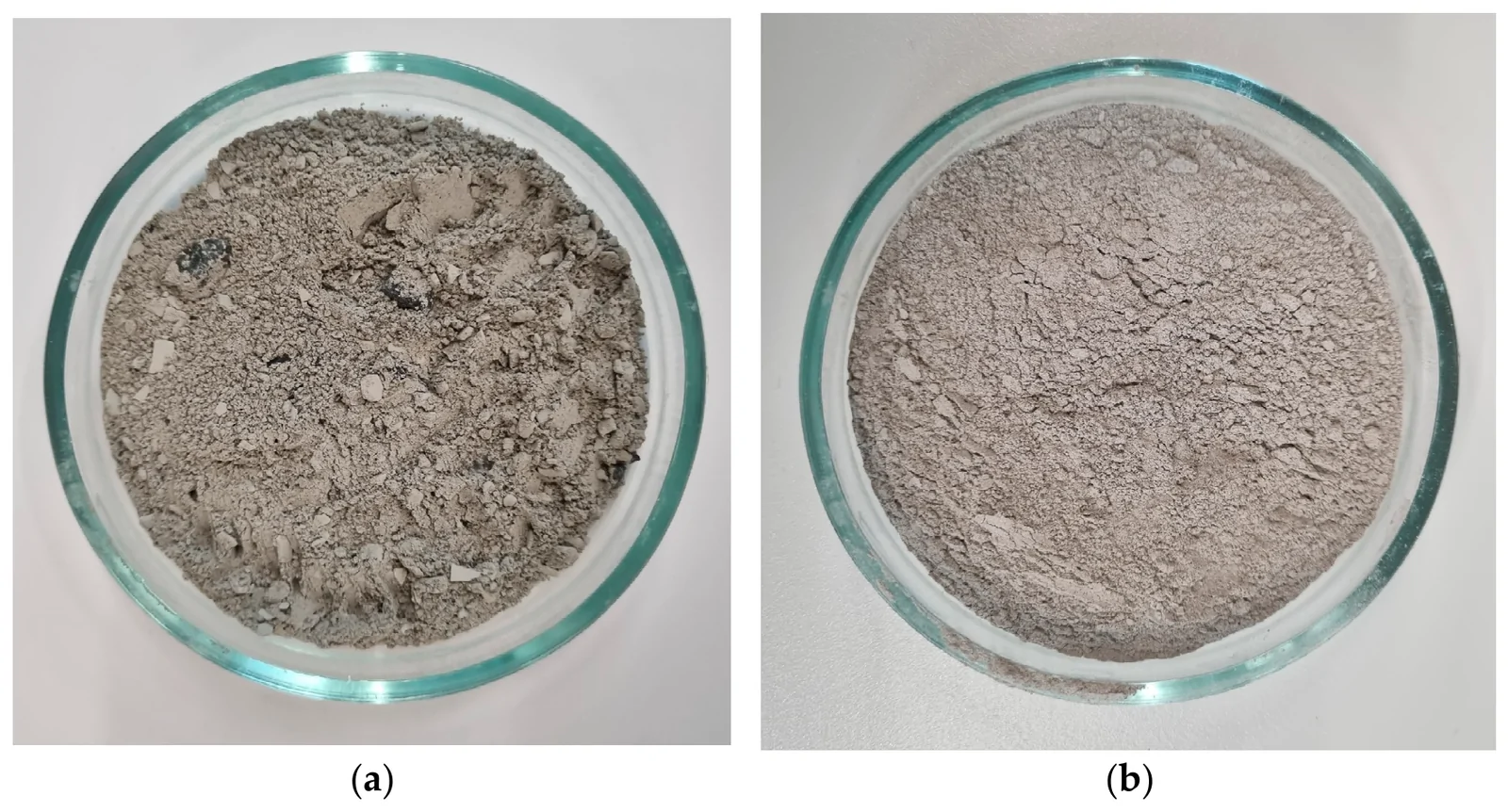
A photograph of VI-75 LR simulant: (**a**) initial powder before sieving; (**b**) <100 µm fraction after sieving.

**Figure 3:**
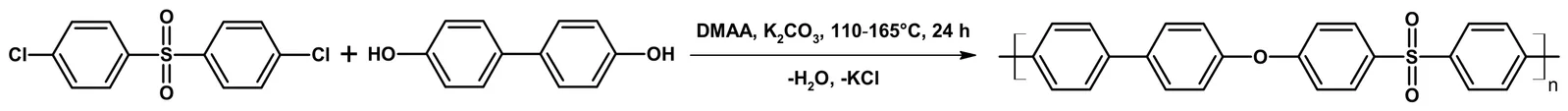
Synthesis scheme of PPSU.

**Figure 4:**
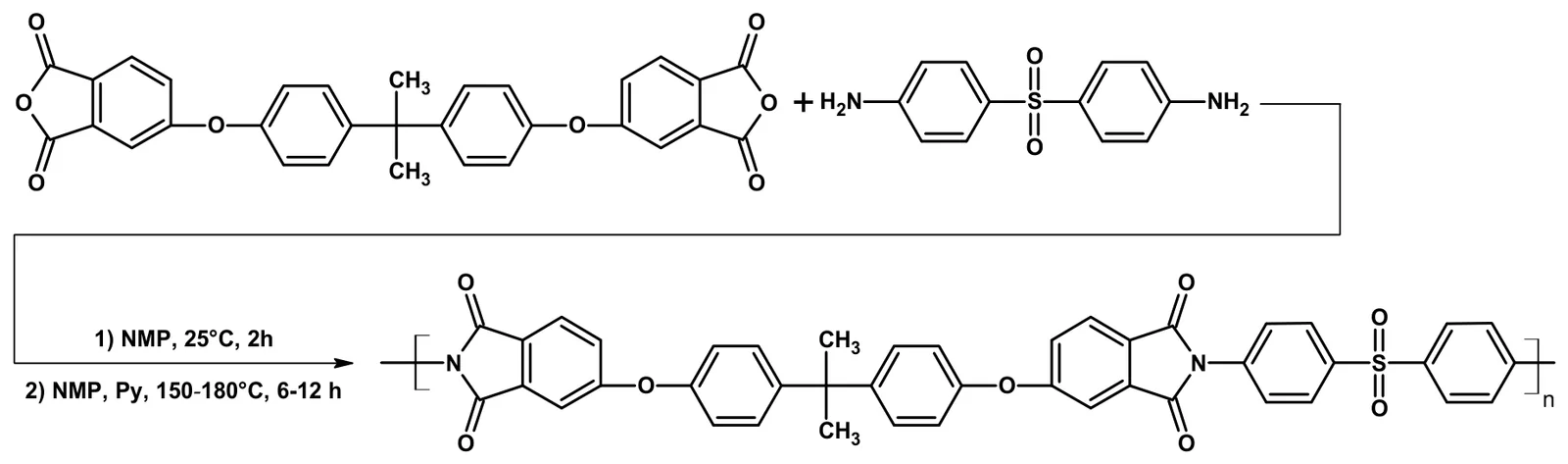
Synthesis scheme of PEI.

**Figure 5:**
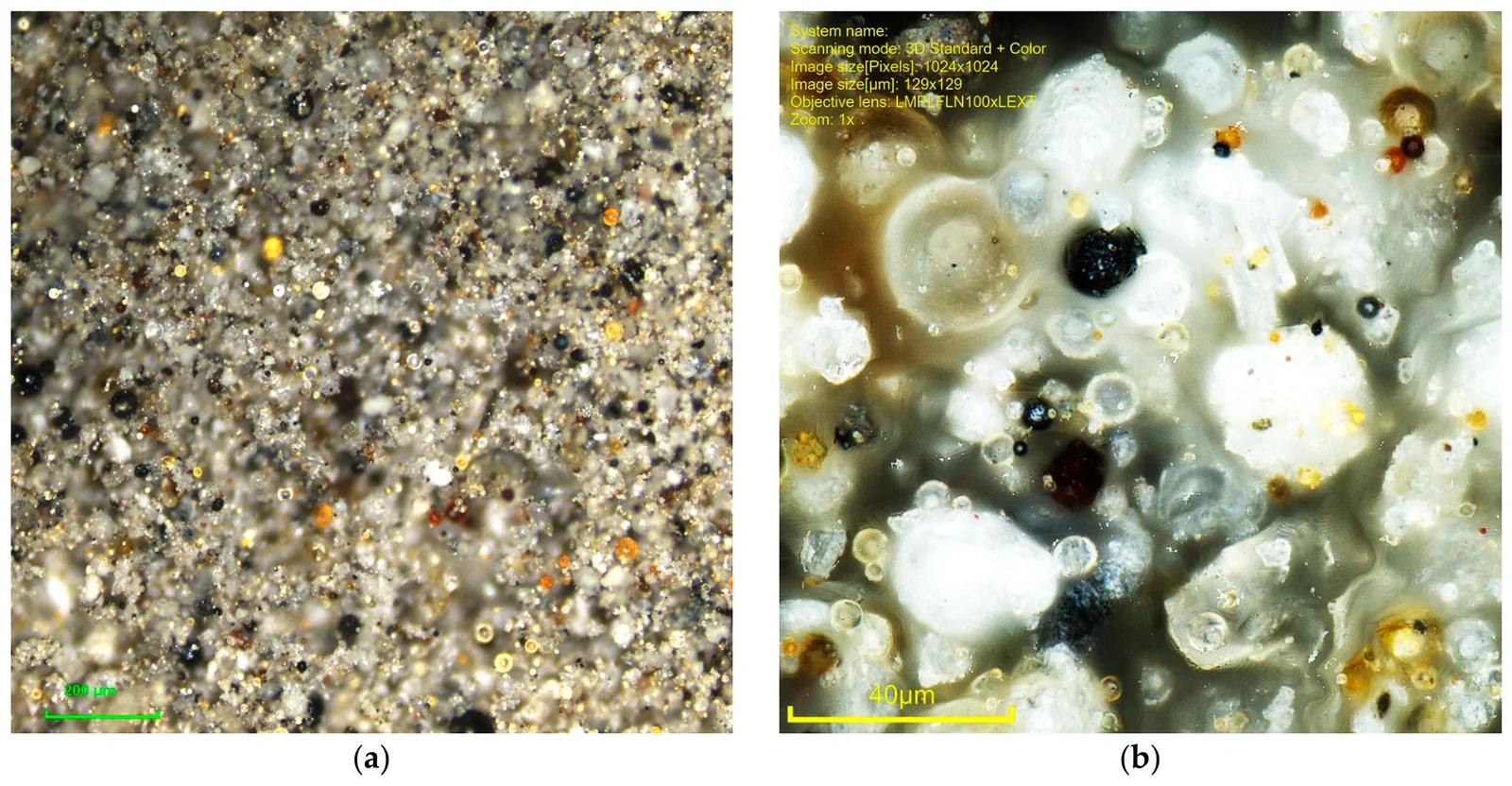
Laser scanning confocal micrographs of the VI-75 LR simulant: (**a**) 20× magnification color image; (**b**) 100× magnification image acquired in 3D Standard + Color mode.

**Figure 6:**
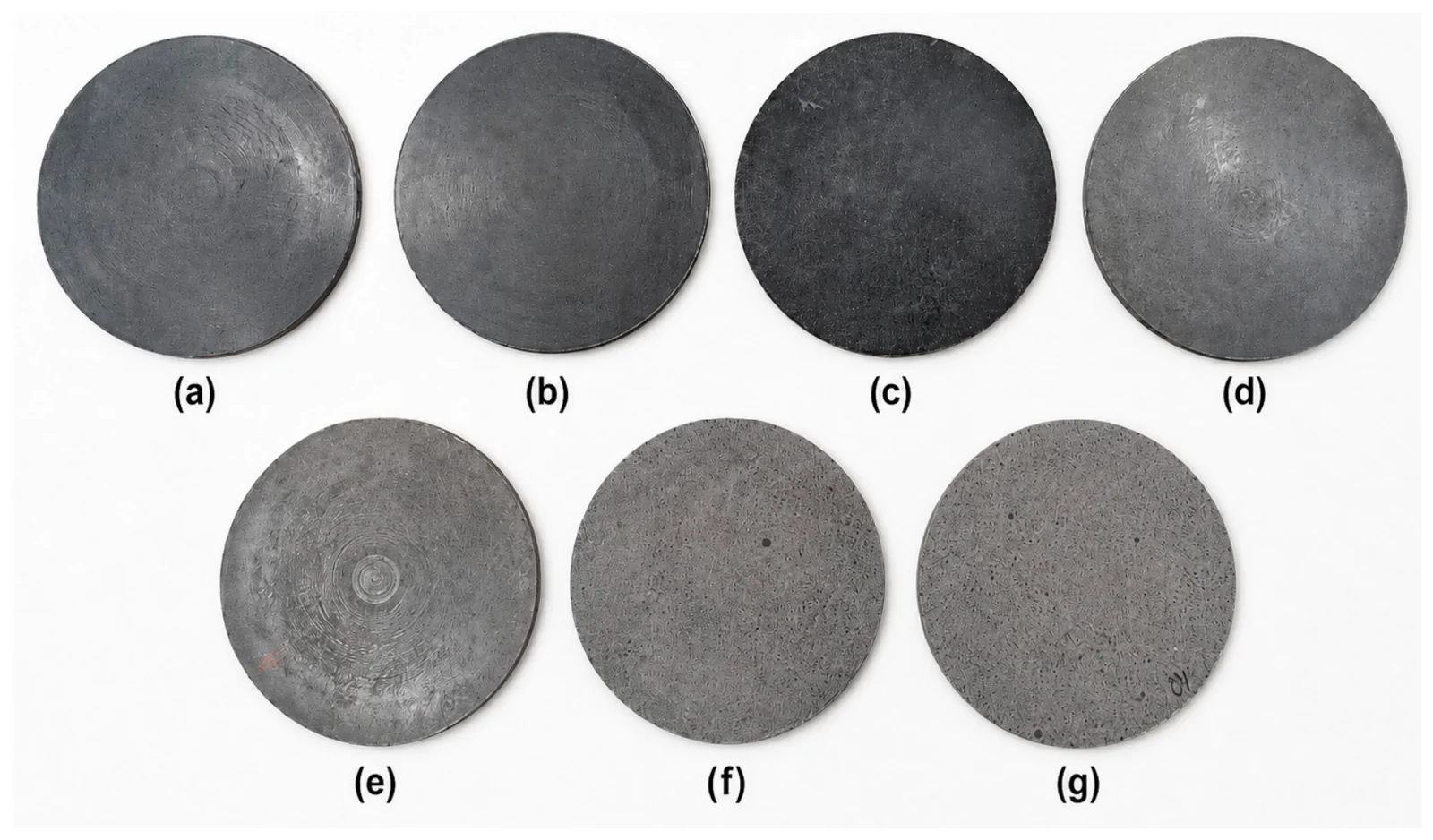
Hot-pressed composite disks with LR simulant/PEI mass ratios: (**a**) 50:50, (**b**) 60:40, (**c**) 70:30, (**d**) 80:20, (**e**) 85:15, (**f**) 90:10, and (**g**) 92.5:7.5 wt.%.

**Figure 7:**
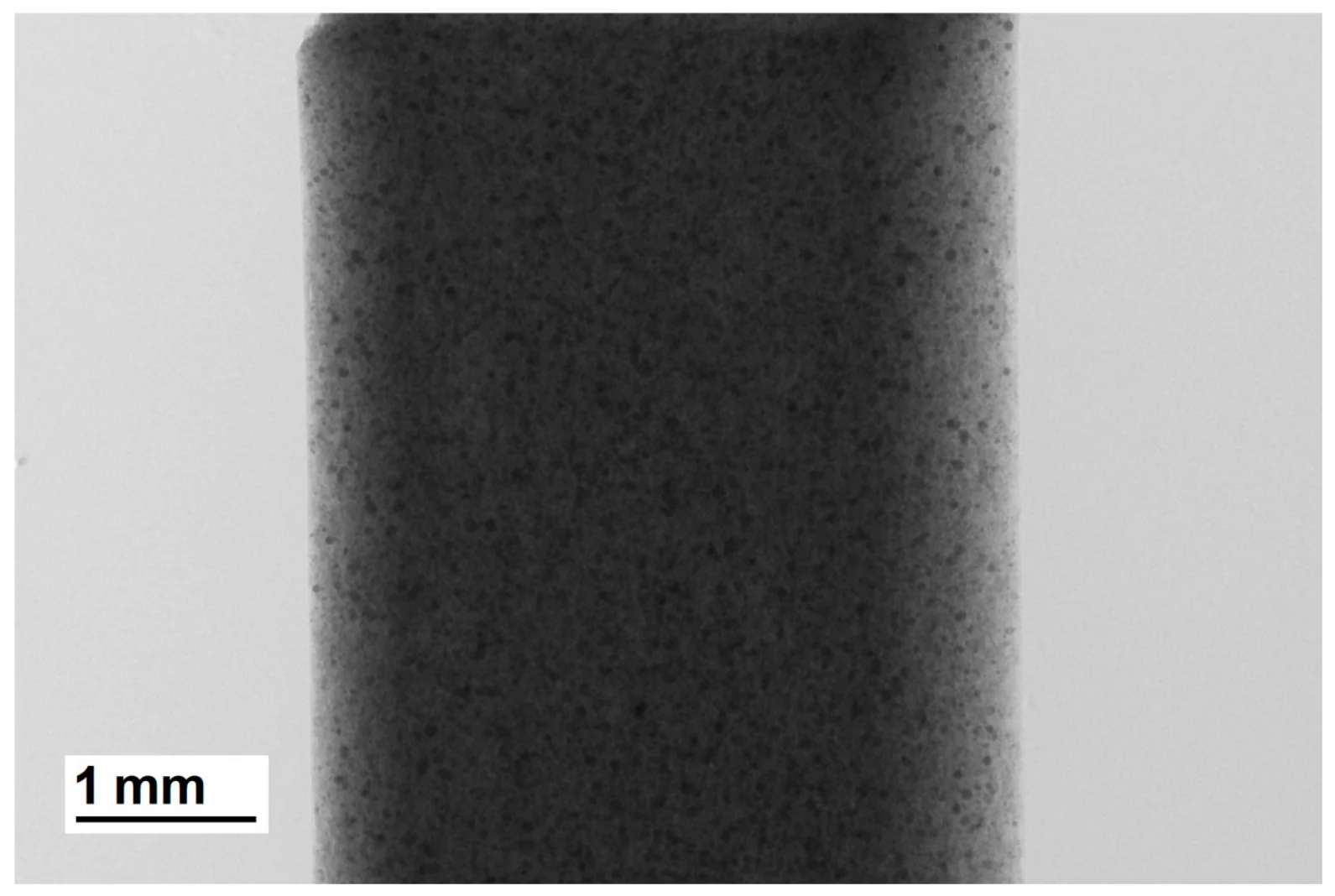
Two-dimensional X-ray projection image of a hot-pressed LR simulant/PEI composite specimen containing 50 wt.% of the filler.

**Figure 8:**
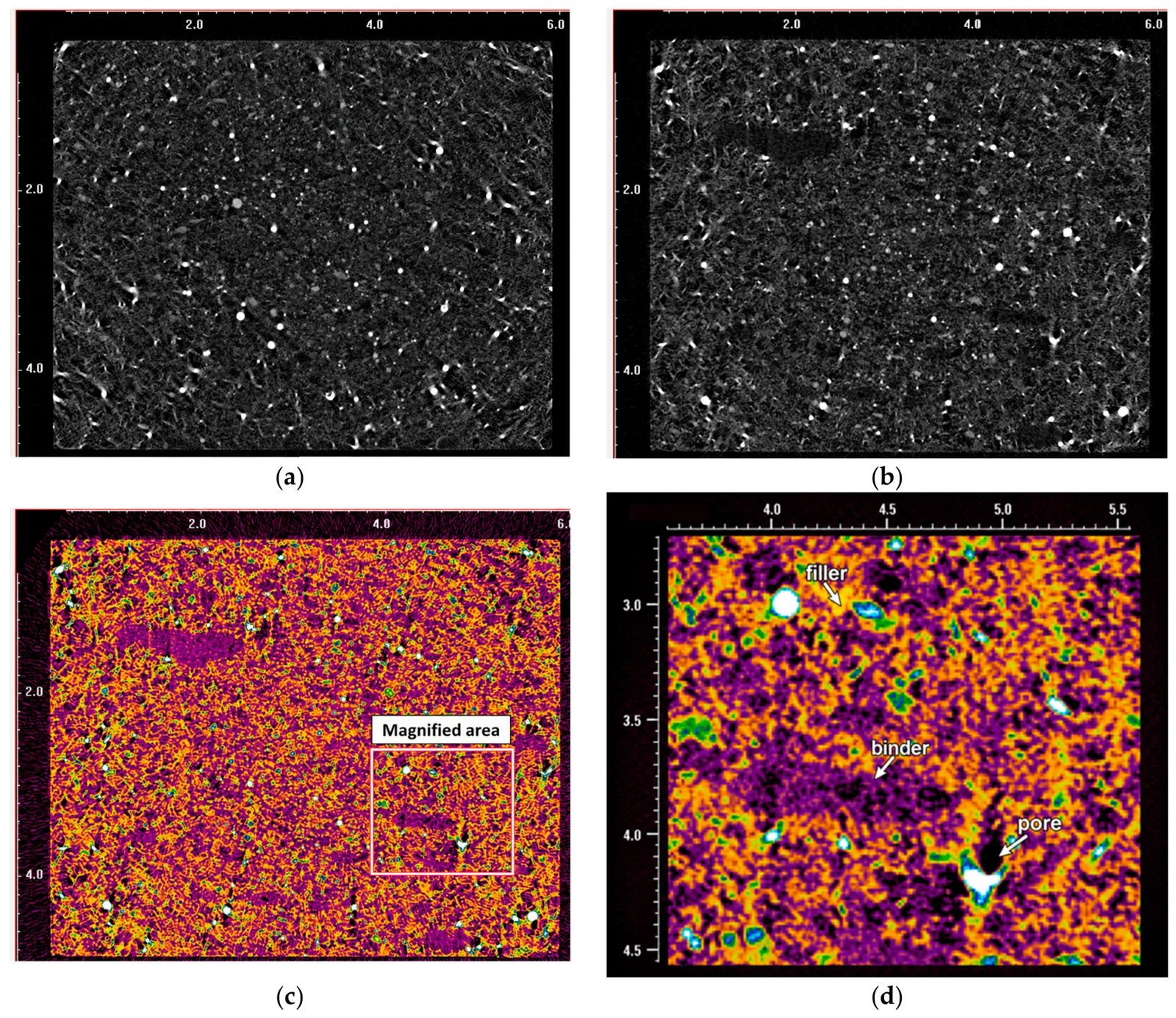
Reconstructed micro-CT images of the LR simulant/PEI composite specimens: (**a**) grayscale representation of a composite containing 50 wt.% of the filler; (**b**) grayscale representation of a composite containing 80 wt.% of the filler; (**c**) false-color rendering of a composite containing 80 wt.% of the filler; (**d**) enlarged view of the selected region showing representative filler, binder, and pore regions.

**Figure 9:**
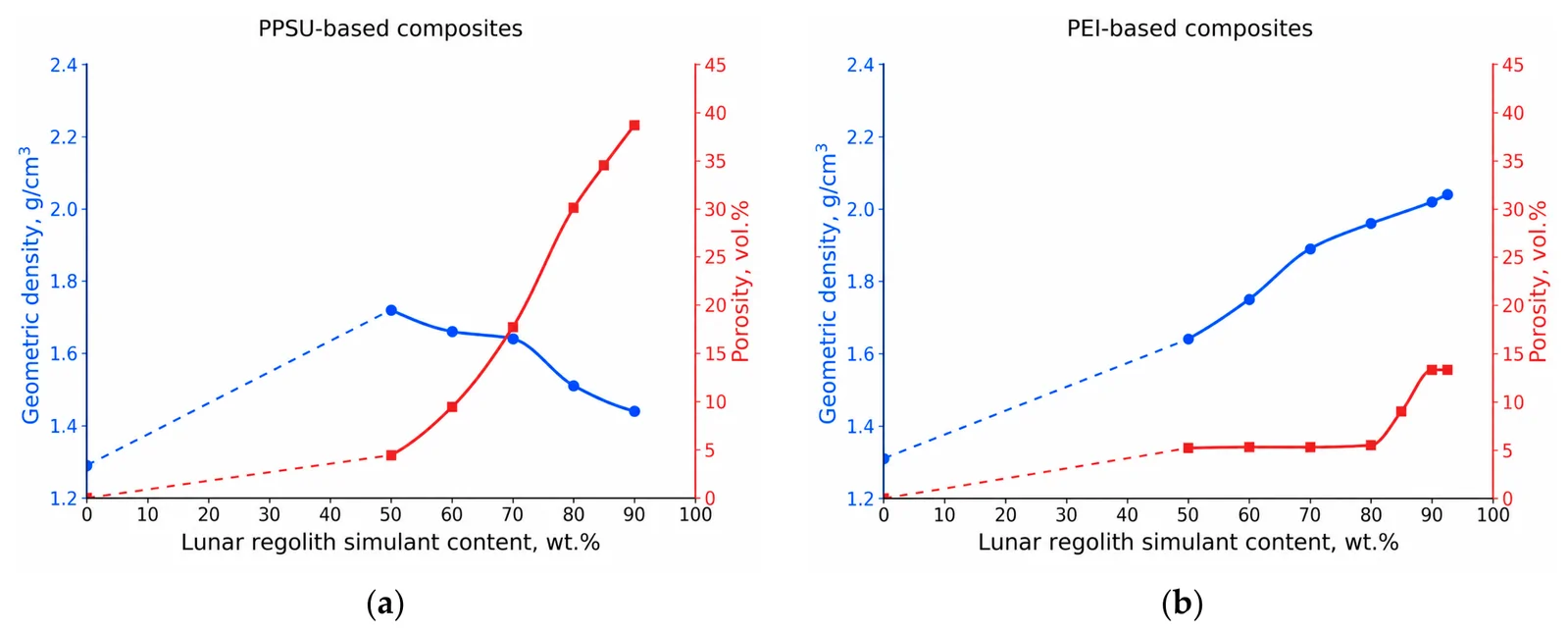
The dependence of geometric density and porosity of LR simulant/thermoplastic composites on the filler content: (**a**) PPSU-based composites and (**b**) PEI-based composites.

**Figure 10:**
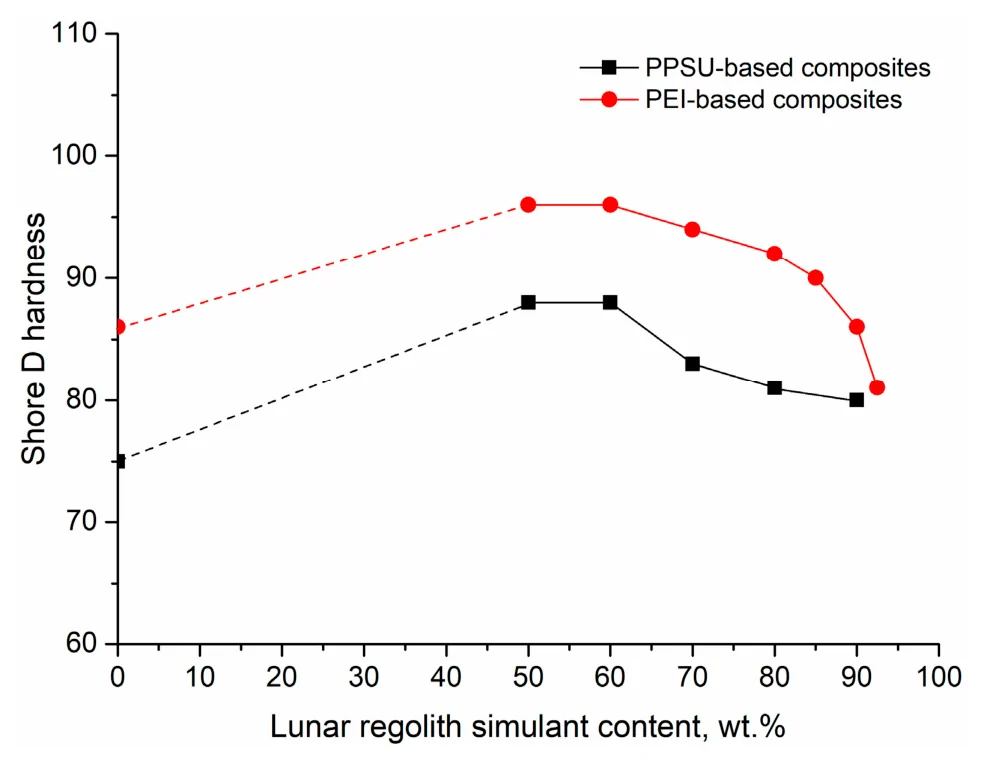
The dependence of Shore D hardness of LR simulant/thermoplastic composites on the filler content.

**Figure 11:**
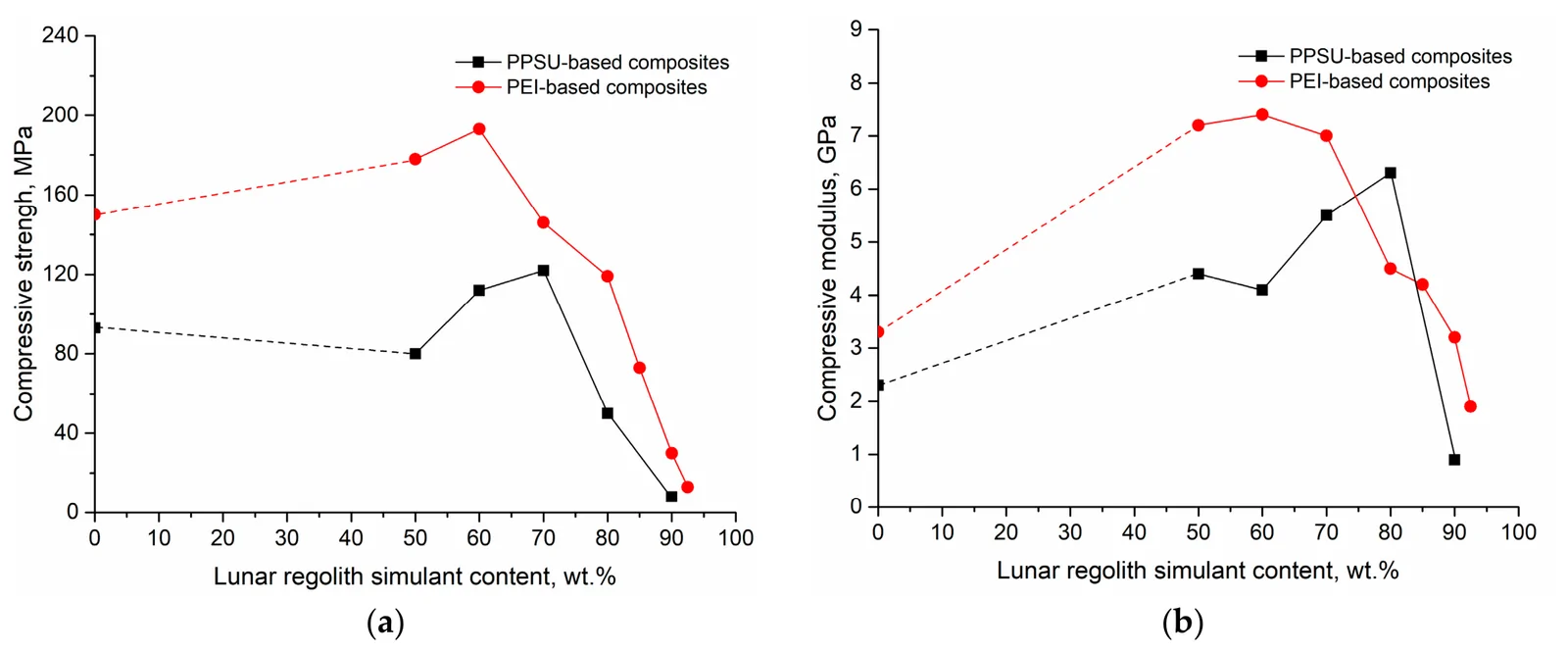
The dependence of compressive mechanical properties of LR simulant/thermoplastic composites on the filler content: (**a**) compressive strength; (**b**) compressive modulus.

**Figure 12:**
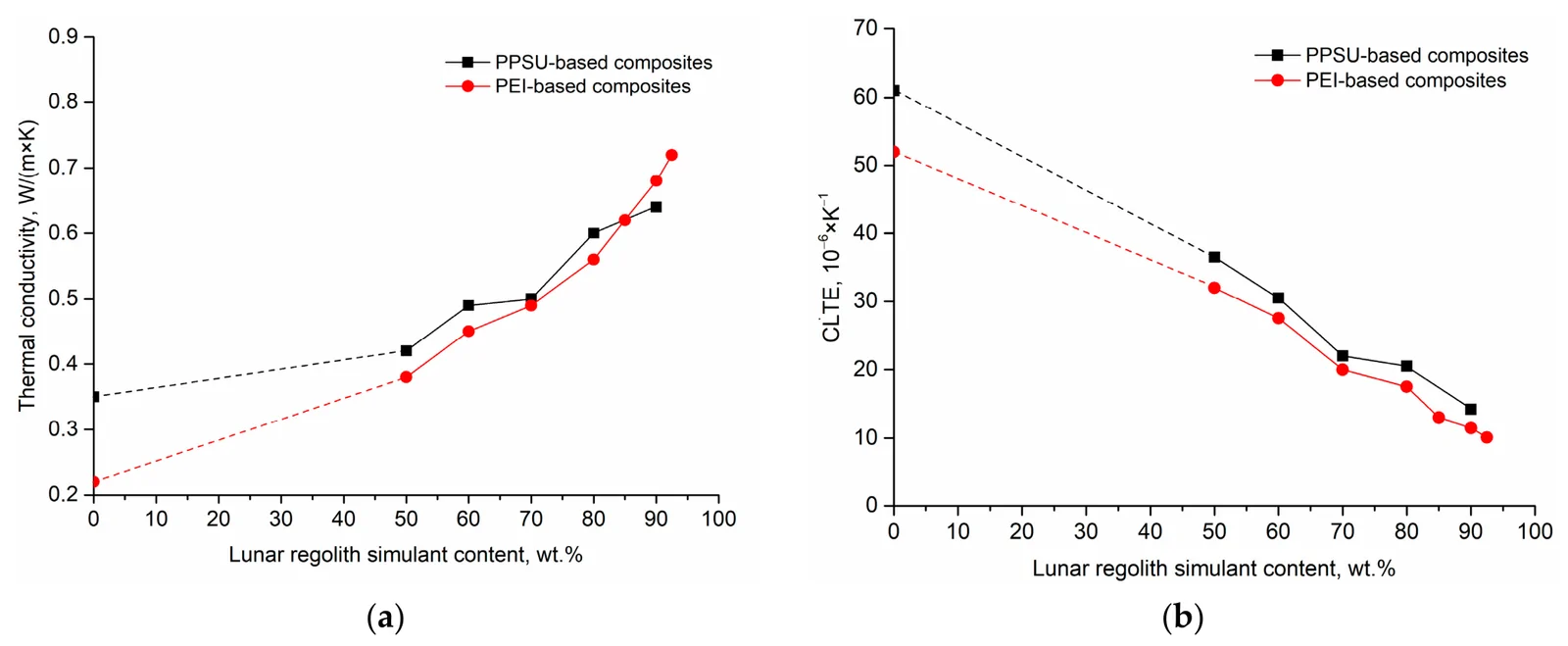
The dependence of thermal properties of LR simulant/thermoplastic composites on the filler content: (**a**) thermal conductivity; (**b**) CLTE.

**Figure 13:**
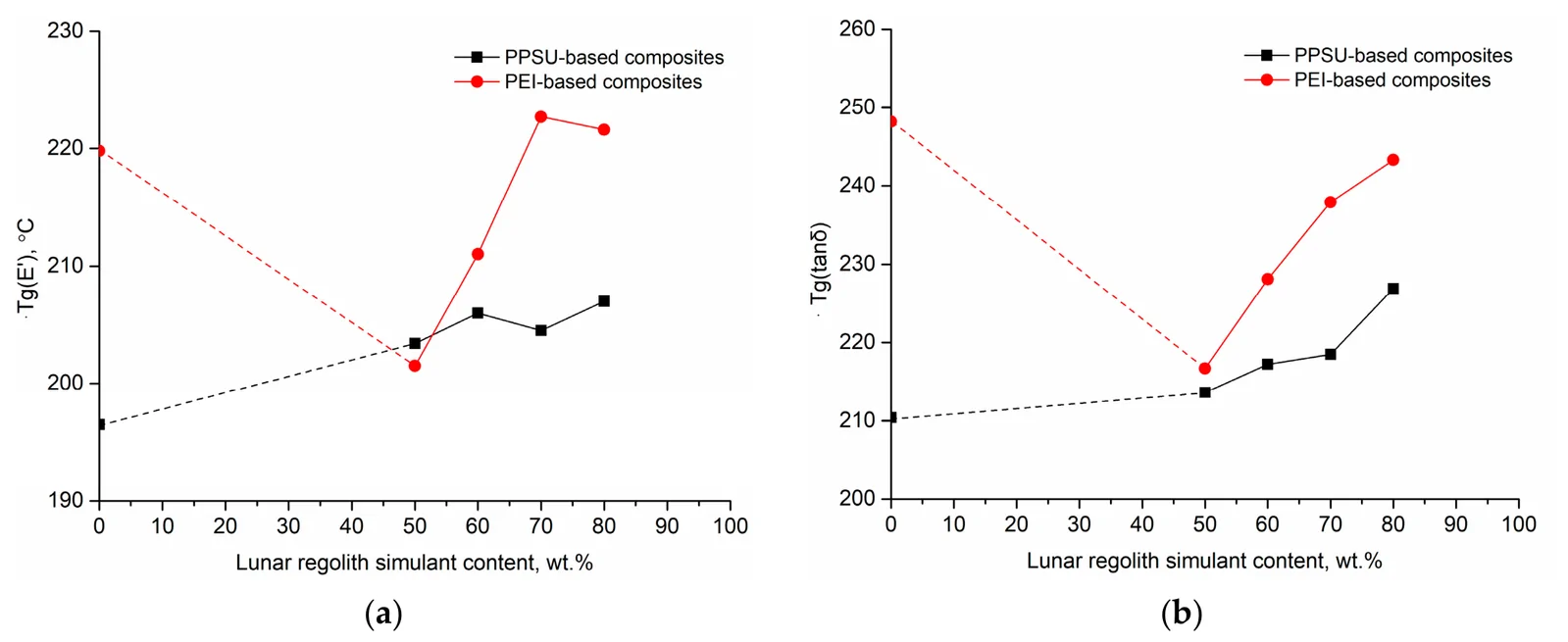
The dependence of T_g_ of LR simulant/thermoplastic composites on the filler content determined by DMA: (**a**) from E′; (**b**) from tan(δ).

**Figure 14:**
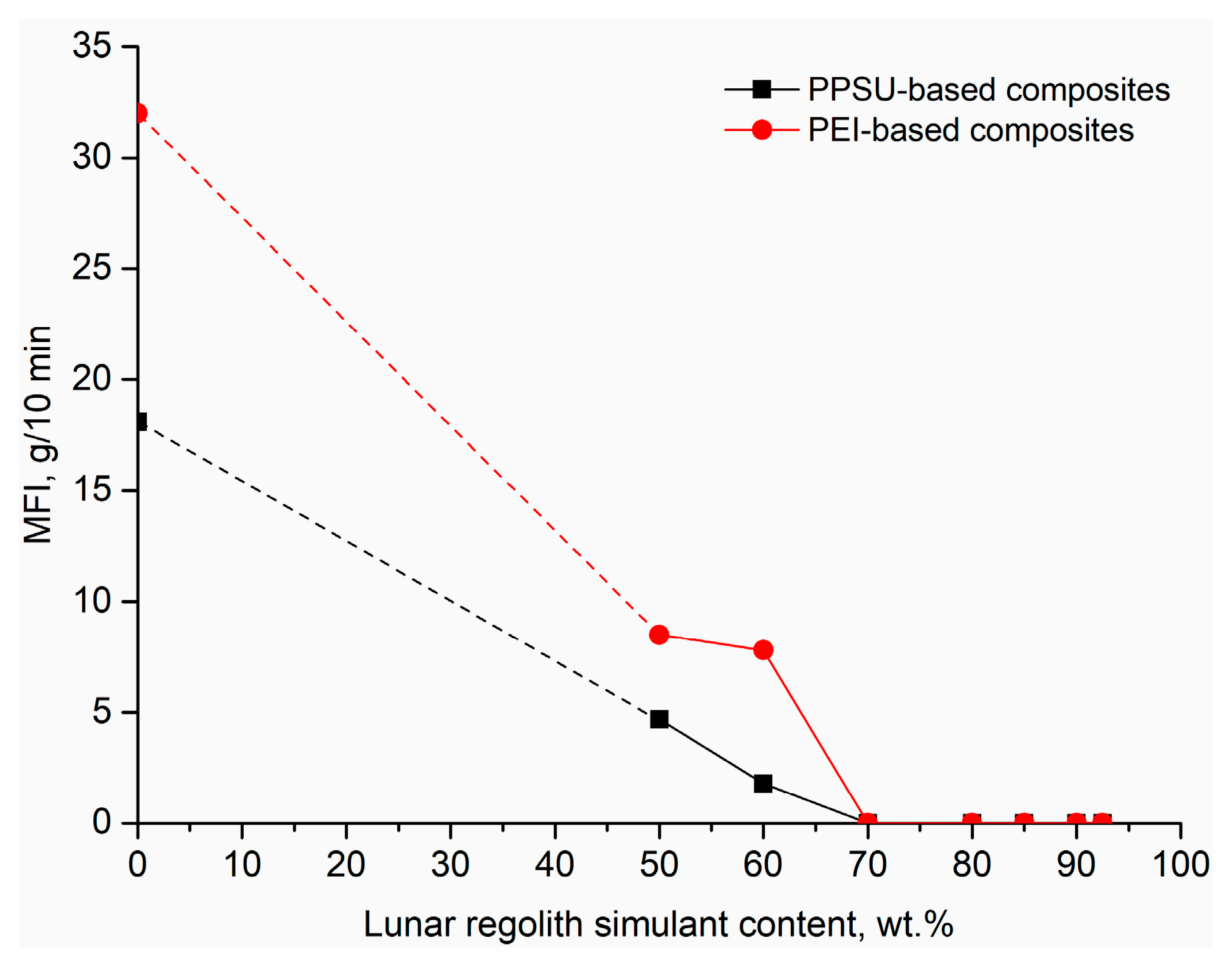
The dependence of the Melt Flow Index of LR simulant/thermoplastic on the filler content; MFI was measured at 340 °C for PPSU and at 370 °C for PEI at a load of 5 kg.

**Table 1:** Chemical composition of LR simulant VI-75.

**Component**	Na_2_O	MgO	Al_2_O_3_	SiO_2_	P_2_O_5_	S	K_2_O	CaO	TiO_2_	MnO	FeO
**Content, wt.%**	0.47	1.7	8.8	68.2	0.09	0.28	0.54	14.43	0.42	0.08	3.63

Major elements are reported as conventional oxide equivalents; sulfur is reported as elemental S, as specified in the material passport.

**Table 2:** LR Simulant VI-75 Particle-Size Distribution.

**Particle–size range, mm**	>0.1	0.1–0.05	0.05–0.01	0.01–0.005	0.005–0.002	<0.002
**Fraction content, wt.%**	0.3	39.0	49.8	8.8	2.0	0.1

**Table 3:** Properties of synthesized PPSU and PEI binders.

Polymer	M_w_, kDa	M_n_, kDa	T_g_, °C	T_5%_, °C	MFI, g/10 min
PPSU	51.4	24.2	219.1	483.1	18.1 *
PEI	55.3	21.3	247.1	510.2	32.0 *

* MFI was measured at 340 °C for PPSU and at 370 °C for PEI at a load of 5 kg.

**Table 4:** Mass and Calculated Volume Fraction of LR Simulant in Obtained Composites.

**LR simulant content, wt.%**	50	60	70	80	85	90	92.5
**LR simulant content in PPSU-based composites,** **vol.%**	33.5	43.0	54.0	66.8	74.1	81.9	−
**LR simulant content in PEI-based composites, ** **vol.%**	33.9	43.4	54.4	67.2	74.4	82.2	86.3

Residual porosity formed during hot pressing was not included in this calculation.

**Table 5:** The compressive strength of LR–based composites with different polymeric binders reported in the literature.

Polymer Binder	LR Content, wt.%	Compressive Strength, MPa	Source
PE	90	12.6–12.9	[27]
PLA	70	43.8–48.9	[83]
	80	46.1–47.9	
	90	43.4–44.9	
PEEK	85	6.0	[46]
	90	16.3	
	95	25.0	
PAEK	80	~100	[84]
	90	~35	
	95	~10	
Epoxy resin	80	119.1	[85]
	84	91.7	
	88	58.5	
	96–99	117–138	[29]
	75	86.4	[31]
	80	98.3	
	85	62.7	
	95	~100	[30]
	98	~26	
	86	9.8 ± 1.1	[86]
	95	44.8	[87]
	96	37.8	
	97	20.5	
	70	~50	[32]
	80	~53	
	90	~42	
	87.5	~100	[88]
	95	~30	
Vitrimer resin	60	96.0	[89]
Potato starch	95.5	71.9–91.7	[90]
Polysaccharide biopolymers	≥98	≤15	[91]

**Table 6:** Summary of experimentally obtained properties in this work and potential application areas of lunar regolith simulant/PEI composites.

LR Simulant Content Range, wt.%	Density, g/cm^3^	Porosity, %	Shore D Hardness	σc, MPa	Ec, GPa	λ, W/(m·K)	CLTE, 10^−6^ K^−1^	MFI, g/10 min	Potential Applications
50–60	1.64– 1.75	5.08– 5.23	96	178–193	7.2– 7.4	0.38– 0.45	27.5– 32.0	7.8–8.5	Melt-based additive manufacturing of complex-geometry parts
70–90	1.89– 2.02	5.23–13.11	86–94	30– 146	3.2– 7.0	0.49– 0.68	11.5– 20.0	0	Hot-pressed load-bearing block-type elements of simple geometry
92.5	2.04	13.28	81	13	1.9	0.72	10.1	0	Hot-pressed simple-shape lightly loaded elements

## Data Availability

The original contributions presented in this study are included in the article and Supplementary Materials. Further inquiries can be directed to the corresponding author.

## Supplementary Materials

The following supporting information can be downloaded at https://www.mdpi.com/article/10.3390/polym18192371/s1. Figure S1. FTIR spectra of the synthesized polymers with the characteristic absorption bands indicated: (a) PPSU and (b) PEI; Figure S2. GPC chromatograms and molecular-weight distributions of synthesized polymers: (a) PPSU and (b) PEI; Figure S3. DSC thermograms of (a) PPSU and (b) PEI; Figure S4. TGA thermograms of (a) PPSU and (b) PEI; Table S1. Compressive strength test results for LR simulant/PPSU composites; Table S2. Compressive strength test results for LR simulant/PEI composites; Figure S5. DMA curves of LR simulant/PPSU composites containing: (a) 50, and (b) 70 wt.% of LR simulant; Figure S6. DMA curves of LR simulant/PEI composites containing: (a) 60, and (b) 80 wt.% of LR simulant.

## Abbreviations

The following abbreviations are used in this manuscript:

ATR	attenuated total reflectance
BPADA	bisphenol A dianhydride
CLTE	coefficient of linear thermal expansion
DCDPS	4,4′-dichlorodiphenyl sulfone
DDS	4,4′-diaminodiphenyl sulfone
DHBP	4,4′-dihydroxybiphenyl
DMA	dynamic mechanical analysis
DMAA	N,N-dimethylacetamide
DMF	N,N-dimethylformamide
DSC	differential scanning calorimetry
FFF	fused filament fabrication
FGF	fused granular fabrication
FTIR	Fourier-transform infrared spectroscopy
GPC	gel permeation chromatography
ISO	International Organization for Standardization
ISRU	in situ resource utilization
LR	lunar regolith
MFI	melt flow index
micro-CT	X-ray micro-computed tomography
NMP	N-methyl-2-pyrrolidone
PAA	polyamic acid
PAEK	polyaryletherketone
PEEK	poly(ether ether ketone)
PEI	polyetherimide
PEKK	poly(ether ketone ketone)
PLA	polylactide
PPSU	polyphenylsulfone
Py	pyridine
TGA	thermogravimetric analysis
