# Supplementary material for: Lunar Regolith Simulant–Filled High-Performance Thermoplastic Composites for Lunar Construction and Their Potential for Additive Manufacturing
Source: Polymers (Basel). 2026 Sep 29;18(19):2371. doi: 10.3390/polym18192371 (PMC13667264; doi:10.3390/polym18192371)
Supplement: Supplementary file 1 [file polymers-18-02371-s001.zip › polymers-4515431-supplementary.pdf]

FTIR

a)

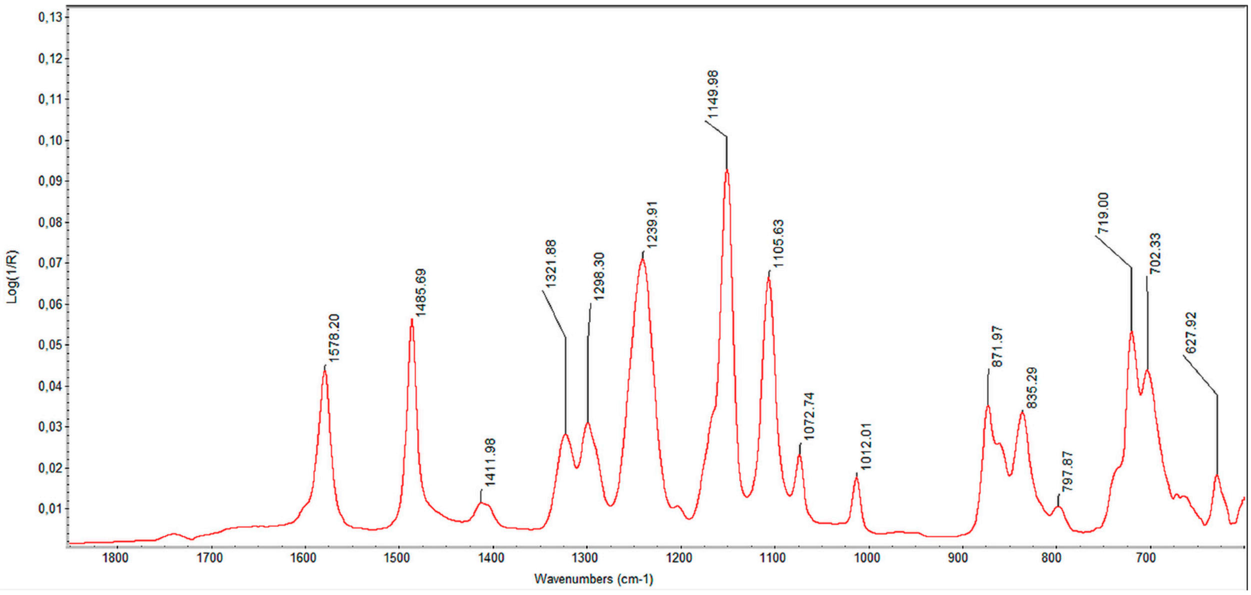

b)

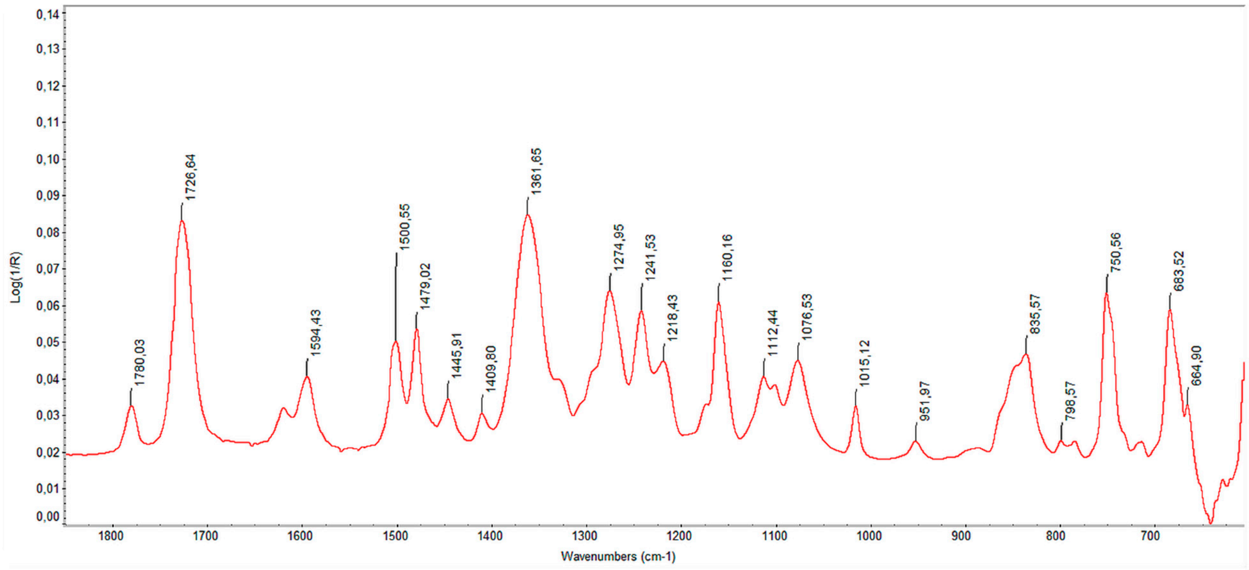

Figure S1. FTIR spectra of the synthesized polymers with the characteristic absorption bands indicated: (a) PPSU and (b) PEI.

FTIR analysis of the samples of synthesized polymers confirms their assignment to the classes of aromatic polyetherimides (PEI) and polyethersulfones (PES).

In the polyphenylsulfone (PPSU) spectrum (Figure S1a), the bands at 1578 and 1486 cm^{-1} correspond to stretching vibrations of the aromatic rings, the intense bands at 1322 and 1149 cm^{-1} are characteristic of the vibrations of the sulfone groups $–SO_2–$, and the absorption band at 1239 cm^{-1} is attributed to stretching vibrations of the aryl ether linkages. The presence of bands in the regions of 1298, 1106, and 835 cm^{-1} is also characteristic of an aromatic polyethersulfone chain.

The spectrum of the PEI (Figure S1b) exhibits intense absorption bands at 1780 and 1727 cm^{-1}, corresponding to the asymmetric and symmetric stretching vibrations, respectively, of

the carbonyl groups in the five-membered imide ring. Characteristic bands are also observed at 1362 cm^{-1}, attributed to C–N–C stretching vibrations, and at 751 cm^{-1}, assigned to deformation vibrations of the imide ring; the band at 1242 cm^{-1} is associated with stretching vibrations of the aryl ether linkages Ar–O–Ar.

Thus, the combination of the identified absorption bands confirms the presence of aromatic, aryl ether, and sulfone structural units in the first sample and fully formed imide rings and aryl ether fragments in the second sample.

GPC

a)

Detector A Channel 1

Peak#	Ret. Time	Area	Height	Conc.	Unit	Mark	Name
1	23,168	1660235	5802	0,000			PPSU
Total		1660235	5802				

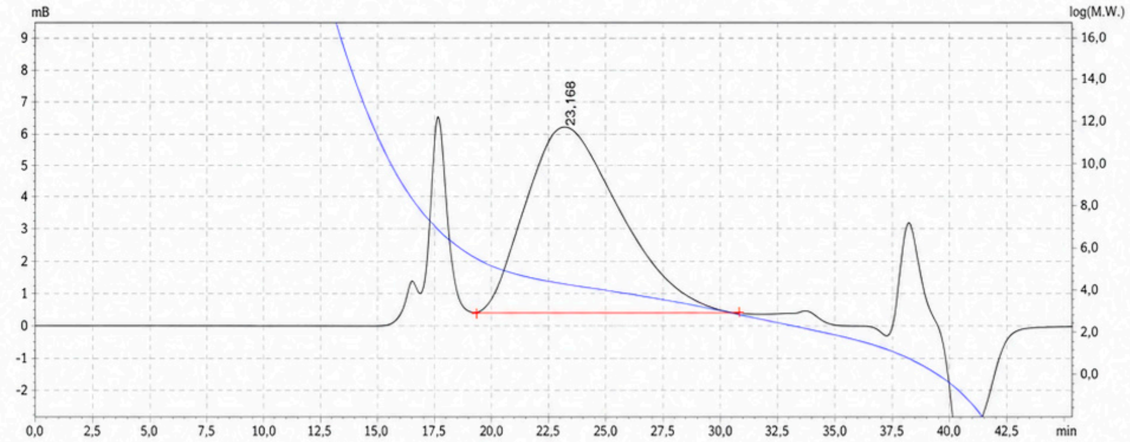

GPC Results

Peak №1 (Detector A Channel 1)
[Average molecular weight]

Number-average molecular weight (Mn)	24244
Weight-average molecular weight (Mw)	51443
Mw/Mn	2,1219

b)

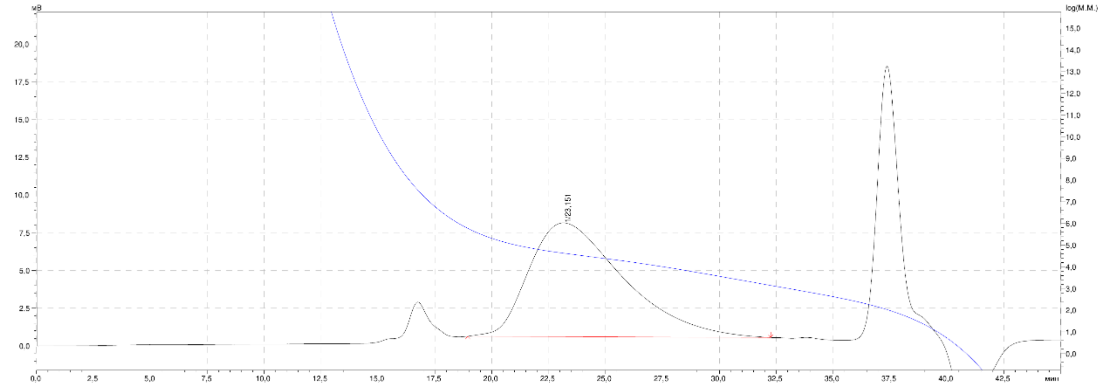

Detector A Channel 1

Peak#	Ret. Time	Area	Height	Conc.	Unit	Mark	Name
1	23,151	2173322	7540	0,000			PEI
Сумма		2173322	7540				

GPC Results

Peak №1 (Detector A Channel 1)
[Average molecular weight]

Number-average molecular weight (Mn)	21306
Weight-average molecular weight (Mw)	55266
Mw/Mn	2,60615

Figure S2. GPC chromatograms and molecular-weight distributions of synthesized polymers: (a) PPSU and (b) PEI.

The GPC chromatograms of both PPSU and PEI contain three characteristic peaks. The first early-eluting peak lies within the exclusion region of the column set and was reproduced in blank injections, indicating an injection-related disturbance; therefore, it was excluded from

integration. The third peak, eluting beyond the total permeation volume, arises from the refractive-index mismatch between the sample solvent and the eluent and carries no molecular-weight information. The second polymer peaks correspond to unimodal molecular-weight distributions for both polymers. PPSU exhibited a number-average molecular weight (M_n) of 24.2 kDa and a weight-average molecular weight (M_w) of 51.4 kDa, with a dispersity ($Đ = M_w/M_n$) of 2.12, which is typical for polymers obtained by step-growth polycondensation. The corresponding values for PEI were 21.3 kDa, 55.3 kDa, and 2.61, respectively.

DSC

a)

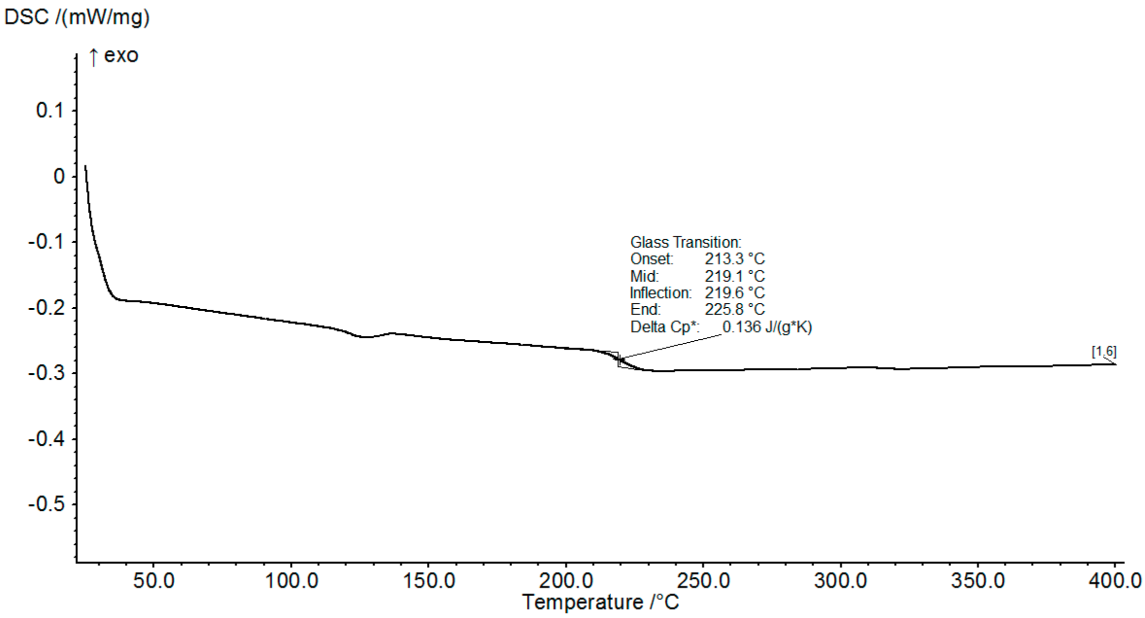

b)

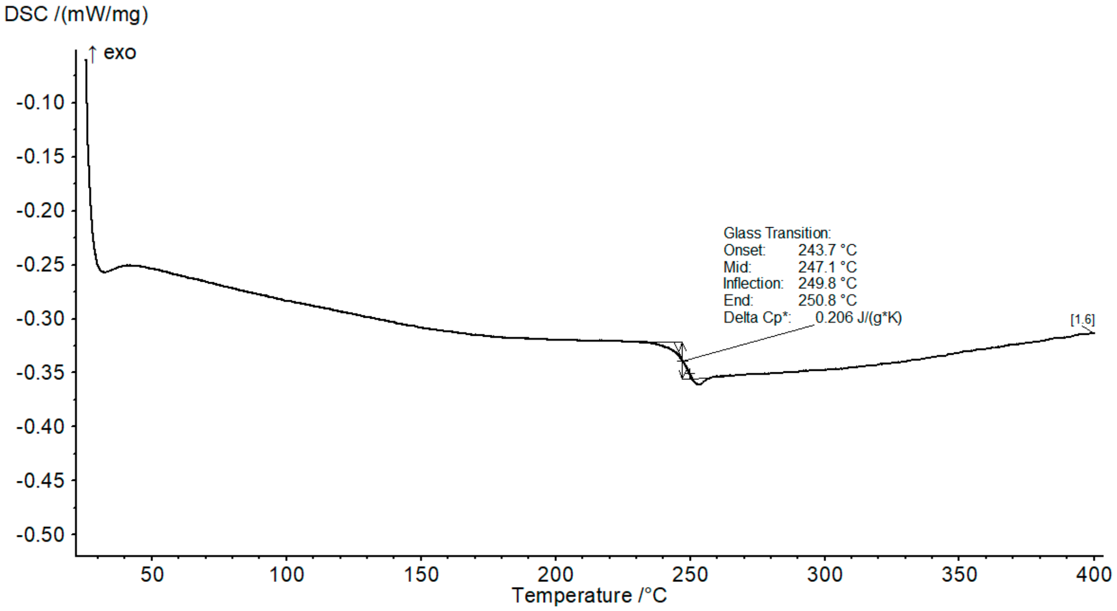

Figure S3. DSC thermograms of (a) PPSU and (b) PEI.

The DSC thermograms of PPSU and PEI exhibited a single endothermic step in the heat-flow signal corresponding to the glass transition, with midpoint temperatures of 219.1°C and 247.1°C, respectively. No exothermic crystallization peaks or endothermic melting peaks were observed over the investigated temperature range, confirming the amorphous nature of both polymers. The higher Tg of PEI can be attributed to the presence of polar imide groups, which enhance intermolecular interactions and restrict segmental mobility of the polymer chains compared with PPSU.

TGA

a)

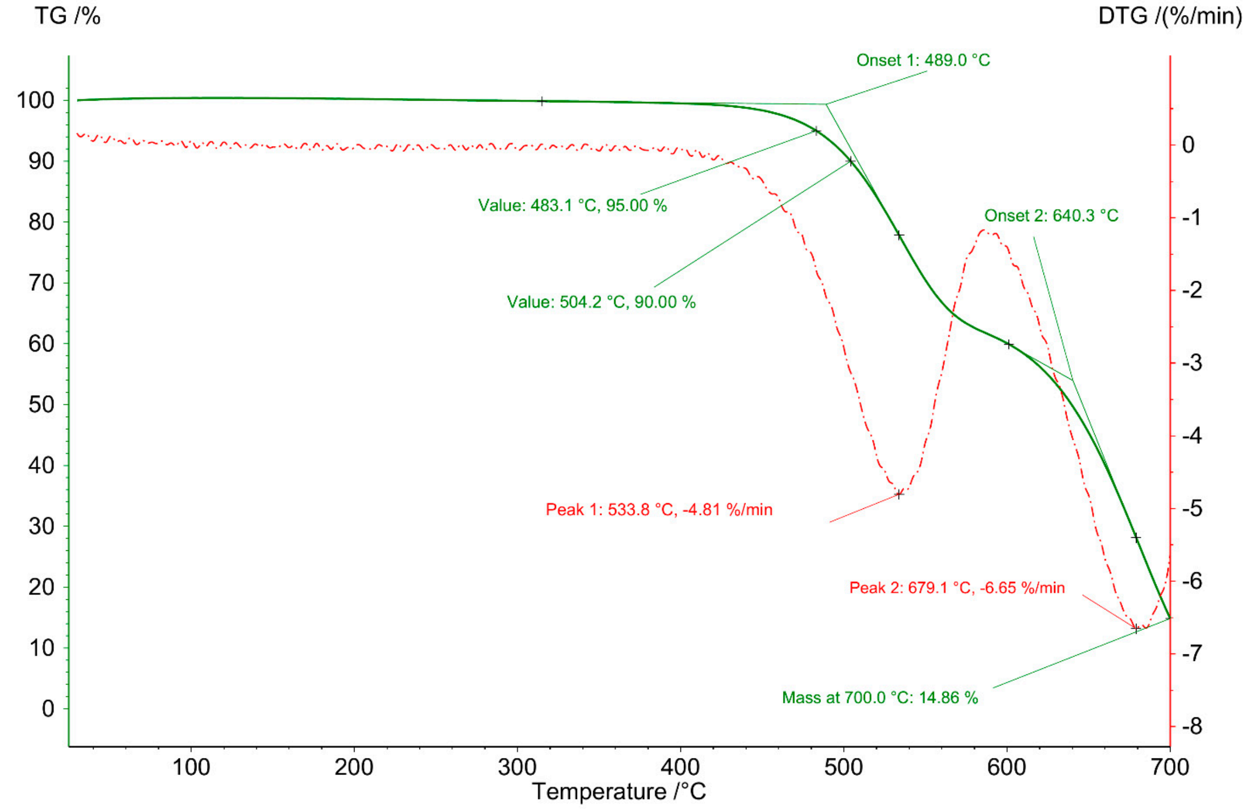

b)

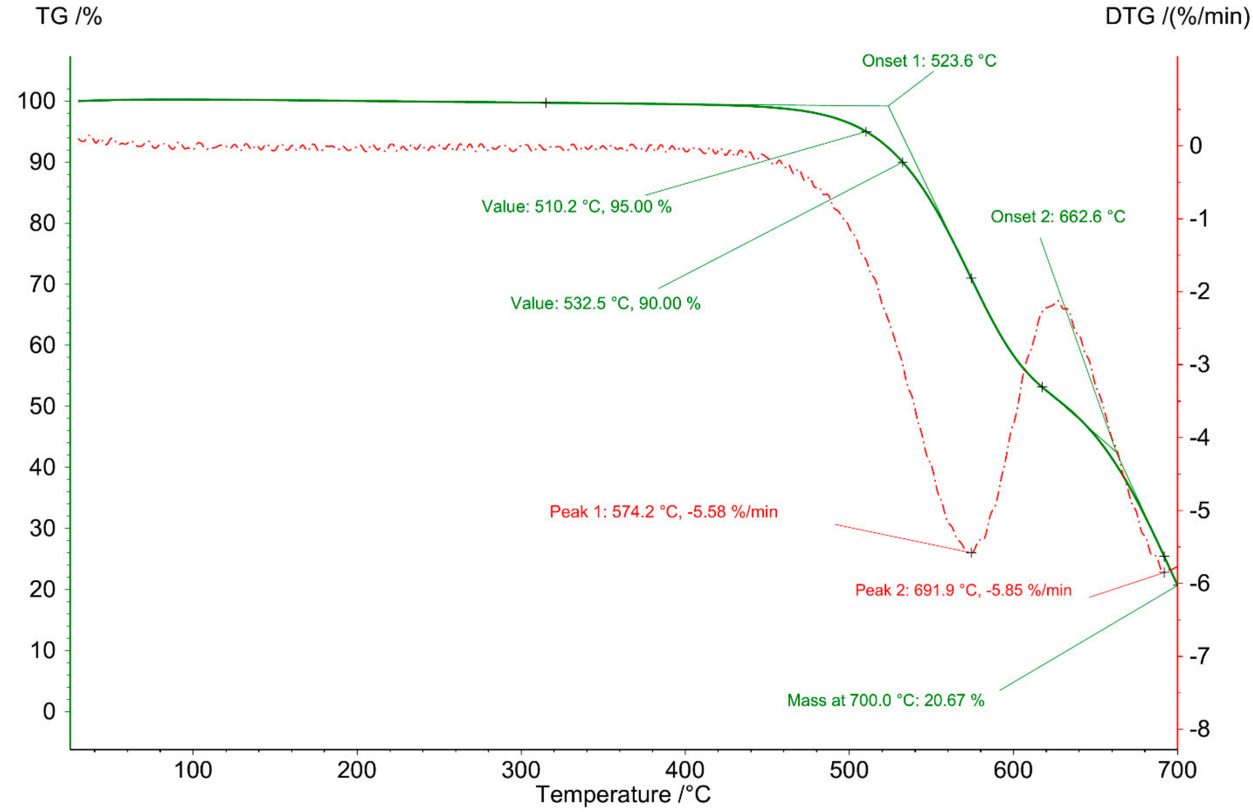

Figure S4. TGA thermograms of (a) PPSU and (b) PEI.

The TGA curves obtained in air show that both PEI and PPSU undergo two-stage thermo-oxidative degradation. The first stage is associated with oxidative scission of the polymer chains and the release of volatile degradation products. After the first stage, a relatively stable carbonaceous residue remains, whose subsequent oxidation produces the second high-temperature mass-loss stage. PEI started to decompose at a higher temperature than PPSU (Onset temperature 1 of 523.6°C for PEI versus 489.0°C for PPSU), indicating greater thermo-oxidative stability. Furthermore, the residual mass at 700°C was higher for PEI (20.67%) than for PPSU (14.86%), suggesting greater stability of the PEI-derived carbonaceous residue.

Mechanical tests

Table S1. Compressive strength test results for LR simulant/PPSU composites

LR, wt.%	σ_1, MPa	σ_2, MPa	σ_3, MPa	σ, MPa	σ_{sd}, MPa
0	98.4	90.9	90.3	93.2	4.5
50	81.1	78.4	79.6	79.7	1.4
60	120.2	102.9	112.5	111.9	8.7
70	124.4	118.9	122.2	121.8	2.8
80	49.4	49.1	52.3	50.3	1.8
90	7.3	7.9	9.1	8.1	0.9

Table S2. Compressive strength test results for LR simulant/PEI composites

LR, wt.%	σ_1, MPa	σ_2, MPa	σ_3, MPa	σ, MPa	σ_{sd}, MPa
0	145.0	146.9	157.8	149.9	6.9
50	178.1	174.5	181.8	178.1	3.7
60	201.2	201.5	177.3	193.3	13.9
70	144.4	149.7	142.7	145.6	3.7
80	123.2	117.7	116.1	119.0	3.7
85	64.1	77.2	77.8	73.0	7.7
90	28.9	29.2	31.7	29.9	1.5
92.5	13.9	12.6	11.6	12.7	1.1

DMA

a)

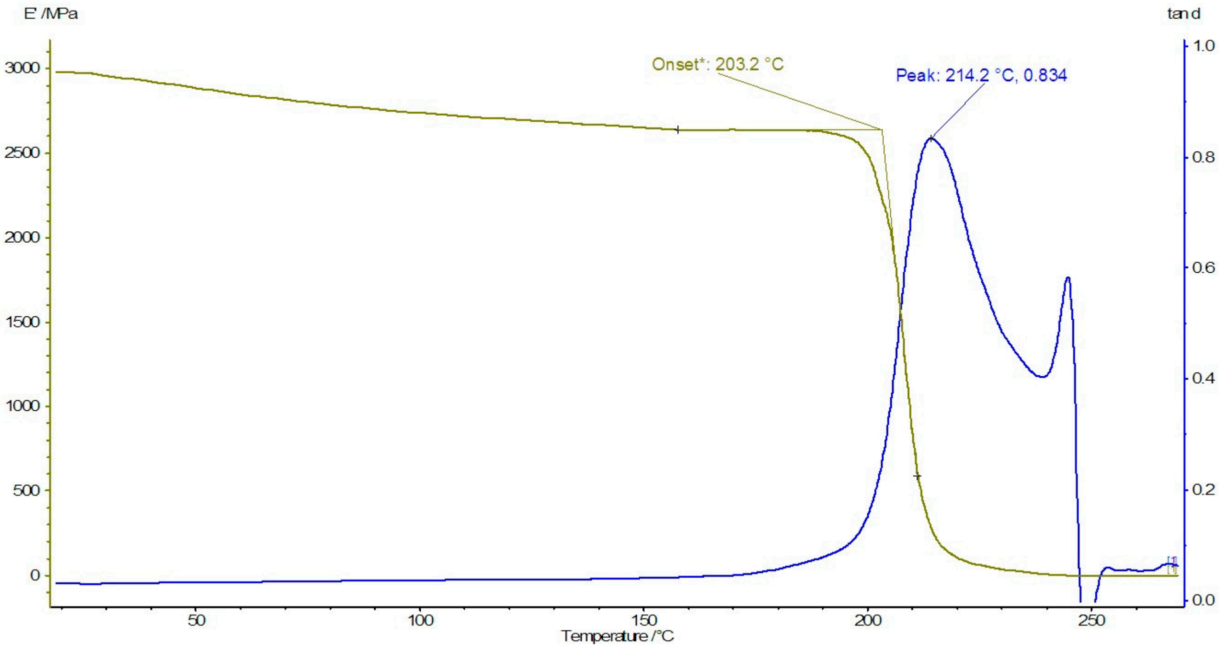

b)

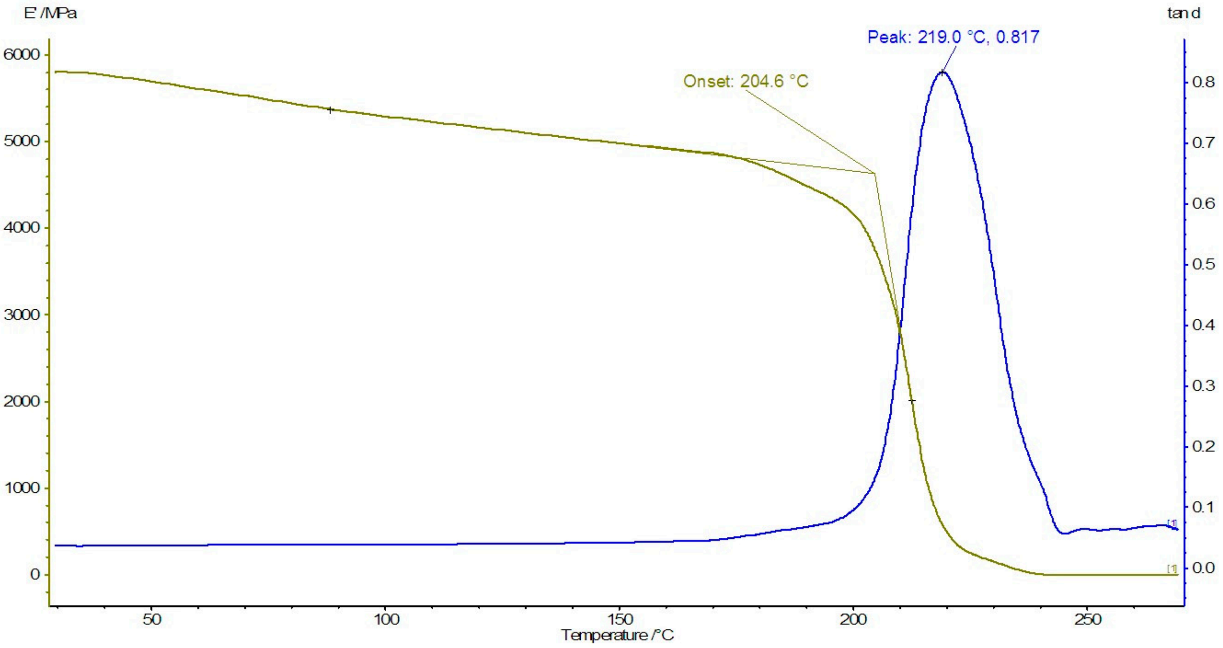

Figure S5. DMA curves of LR simulant/PPSU composites containing: (a) 50, and (b) 70 wt.% of LR simulant.

a)

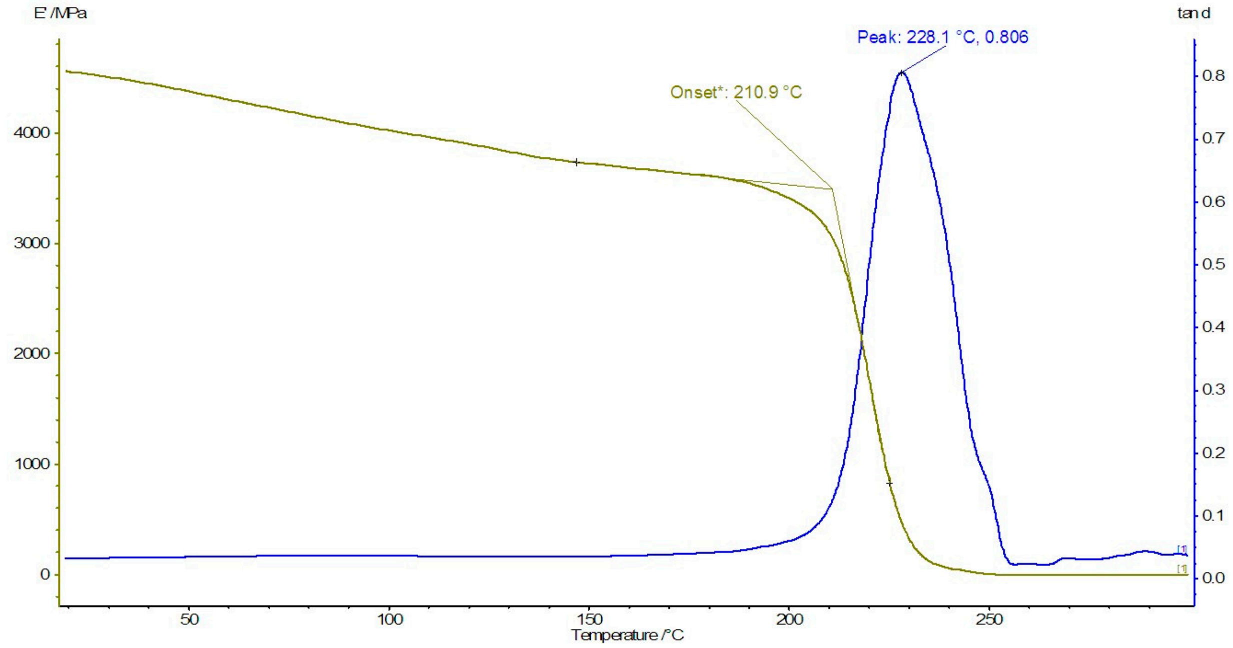

b)

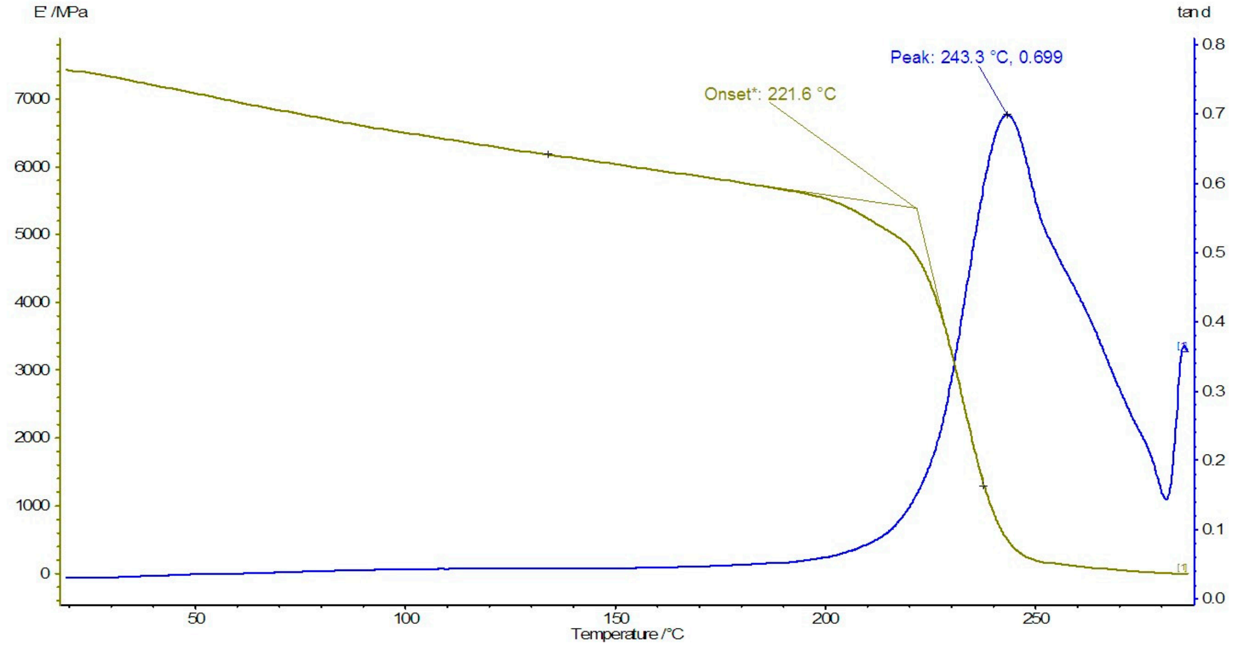

Figure S6. DMA curves of LR simulant/PEI composites containing: (a) 60, and (b) 80 wt.% of LR simulant.
